# Targeted photoswitchable imaging of intracellular glutathione by a photochromic glycosheet sensor

**DOI:** 10.3762/bjoc.15.230

**Published:** 2019-10-07

**Authors:** Xianzhi Chai, Hai-Hao Han, Yi Zang, Jia Li, Xiao-Peng He, Junji Zhang, He Tian

**Affiliations:** 1 Key Laboratory for Advanced Materials and Joint International Research Laboratory of Precision Chemistry and Molecular Engineering, Feringa Nobel Prize Scientist Joint Research Center, School of Chemistry and Molecular Engineering, East China University of Science and Technology, 130 Meilong Road, Shanghai 200237, People’s Republic of China; 2 National Center for Drug Screening, State Key Laboratory of Drug Research, Shanghai Institute of Materia Medica, Chinese Academy of Sciences, 189 Guo Shoujing Rd., Shanghai 201203, People’s Republic of China

**Keywords:** intracellular GSH, molecular switches, photochromic glycosheet, photoswitchable imaging, 2D MnO_2_ nanosheets

## Abstract

The development of photochromic fluorescence sensors with dynamic and multiple-signaling is beneficial to the improvement of biosensing/imaging precision. However, elaborate designs with complicated molecular structures are always required to integrate these functions into one molecule. By taking advantages of both redox-active/high loading features of two-dimensional (2D) manganese dioxide (MnO_2_) and dynamic fluorescence photoswitching of photochromic sensors, we here design a hybrid photochromic MnO_2_ glycosheet (**Glyco-DTE@MnO_2_**) to achieve the photoswitchable imaging of intracellular glutathione (GSH). The photochromic glycosheet manifests significantly turn-on fluorescence and dynamic ON/OFF fluorescence signals in response to GSH, which makes it favorable for intracellular GSH double-check in targeted human hepatoma cell line (HepG2) through the recognition between β-D-galactoside and asialoglycoprotein receptor (ASGPr) on cell membranes. The dynamic fluorescence signals and excellent selectivity for detection and imaging of GSH ensure the precise determination of cell states, promoting its potential applications in future disease diagnosis and therapy.

## Introduction

Cells are the basic structure and functional unit of biological organisms. Human diseases and aging are closely related to the states of cells. Thorough understanding of intracellular signal transduction and metabolic processes may provide great opportunities for early disease diagnosis and treatment. To achieve this goal, cell-imaging with fluorescence sensors becomes a booming research field since it enables the high-resolution visualization of intracellular activities [1–2]. Nonetheless, conventional fluorescence sensors always encounter background signal interferences during cell imaging, which are usually generated from bioluminescence and light scattering in the intracellular environment [3]. This may lead to the deviation in judging the morphology and state of the cells, e.g., causing false-positive/negative results. Generally, strategies like designing ratiometric [4–5] or near-infrared [6–8] fluorescence sensors are applied to overcome this obstacle. Recently, a novel category of photochromic probes with light-controlled dynamic fluorescence signals has been developed, aiming at reducing interferences and improving sensing precision in complex physiological environments [9–17]. This photoresponsive design presents several advantages over conventional probes: 1) The light-activation mode endows the probe with light-controllable “ON/OFF” working states. The OFF-state (one of the photoisomer or photocaged structure) “masks” the probe before reaching the target analyte, avoiding unwanted interactions with other abundant species in the “working zone”, or unnecessary consumption with analyte in nontargeted locations during in vivo/intracellular transport [18–20]. 2) A dynamic ON/OFF fluorescence signal is generated for reversible imaging of a targeted analyte (termed as “double-check”), which can facilitate a better discrimination of the analyte signal from the background interferences [9–11]. As a result, more precise outputs can be obtained for targeted analytes even at low concentrations.

**Scheme 1:**
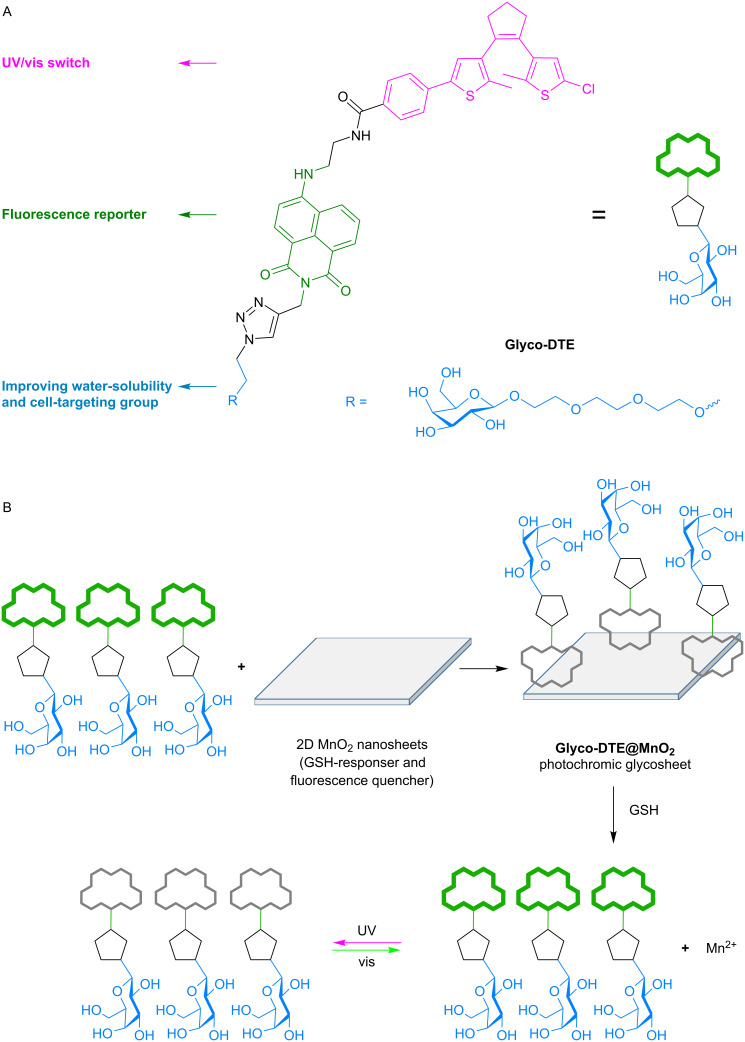
The structure (A) of reporter **Glyco-DTE** and working principle (B) of photochromic glycosheet **Glyco-DTE@MnO_2_** for targeted detection and imaging of GSH in HepG2 cells.

Though promising, common “photochromophore–fluorophore”-type sensors require elaborate designs to integrate multifunctionality (e.g., photoswitching, fluorescence sensing, targetability, water solubility, etc.) into one molecule that could be accessible to various biosensing scenarios and ensure the imaging precision. This might cause limitations in further applications as complicated structures may lead to unpredictable performances and high cost that are not suitable for future commercialization. To simplify the sensor design and further broaden the availability of photoswitchable biosensing, herein we report a glycosheet hybrid sensor (**Glyco-DTE@MnO_2_**) fabricated by 2D MnO_2_ nanosheets and dithienylethene fluorescence reporter (**Glyco-DTE**) to achieve cell-targeted photoswitchable imaging of intracellular GSH. As shown in Scheme 1, **Glyco-DTE@MnO_2_** glycosheets were formed by assembling **Glyco-DTE** onto the surface of 2D MnO_2_ nanosheets, which quench the fluorescence from **Glyco-DTE** reporter. Recent studies discovered that 2D MnO_2_ nanosheets tend to undergo a facile reduction with GSH, MnO_2_ + 2GSH + 2H^+^ → Mn^2+^ + GSSG + 2H_2_O [21], and be degraded into Mn^2+^ that revives initial fluorescence signals. Furthermore, the generated Mn^2+^ can also perform as potential trigger for sequential functions, e.g., DNAzymes [22]. These interesting performances promote MnO_2_ nanosheets as promising candidate for various physiological applications as biosensing/imaging, bioactivity modulation, drug delivery, etc [23–24].

In our system, the **Glyco-DTE@MnO_2_** hybrid sensor undergoes decomposition when encountering the overexpressed intracellular GSH in HepG2 cell lines, following the recovery of the photoswitchable fluorescence signal regulated by **Glyco-DTE**. More importantly, the β-ᴅ-galactoside cell-targeting moiety linked with **Glyco-DTE** forms a glyco-array on the MnO_2_ nanosheets that not only enhances the water solubility but also the cell targetability of the hybrid system towards HepG2 cell lines [25–26]. Therefore, by simply incorporating the photochromic fluorescence reporter into GSH-responsive MnO_2_ nanosheets, a highly efficient photoswitchable hybrid biosensor is successfully presented with the demanded functionality for precise cell imaging.

## Results and Discussion

### Synthesis of dithienylethene fluorescence reporter (Glyco-DTE)

**Scheme 2:**
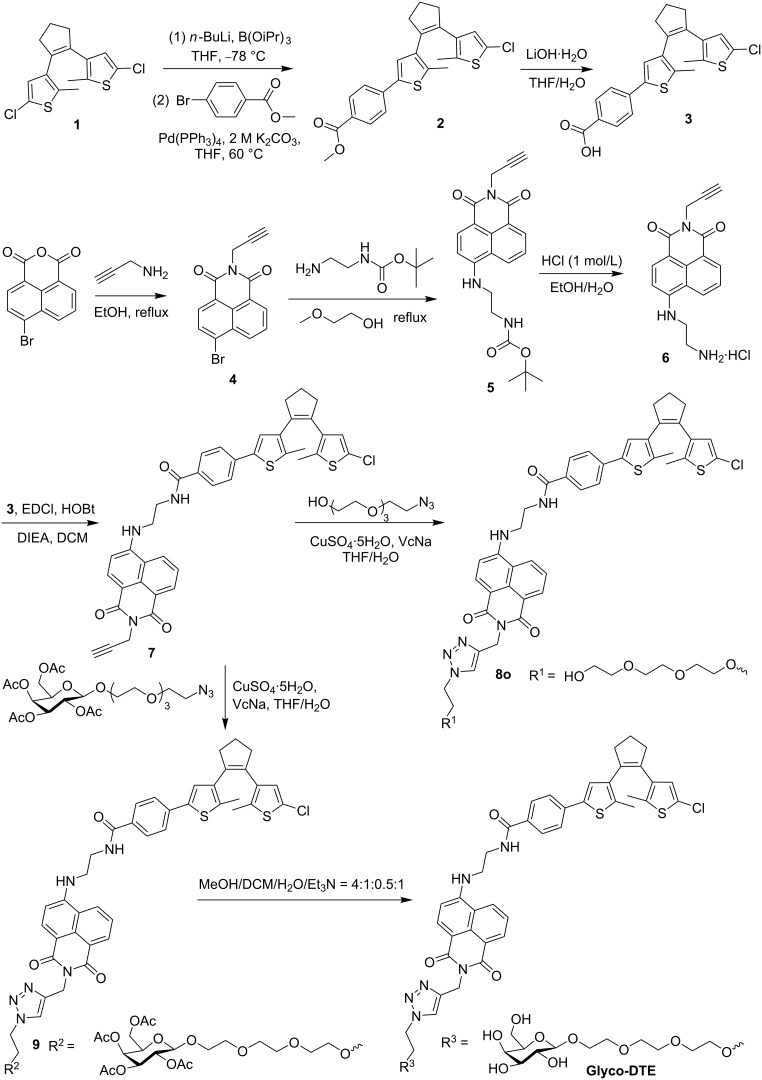
Synthetic route to dithienylethene fluorescence reporters **Glyco-DTE** and **8o**. VcNa: sodium ascorbate.

The synthesis of dithienylethene fluorescence reporter **Glyco-DTE** is shown in Scheme 2. The dithienylethene derivative **3** was prepared by Suzuki coupling of dithienylethene **1** with methyl 4-bromobenzoate followed by hydrolysis with lithium hydroxide. The naphthalimide fluorophore **6** was synthesized through bromide **4** according to reported methods [27]. Then, the photochromic fluorophore intermediate **7** was synthesized by coupling compounds **3** and **6** through amidation. The **Glyco-DTE** reporter was prepared by click reaction between compound **7** and acetylated β-ᴅ-galactoside, followed by deacetylation. Similarly, a control reporter **8o** with a PEG chain instead of the galactoside targeting group was also prepared. The detailed synthetic procedures and characterizations are given in Supporting Information File 1.

### Photochromic performances of Glyco-DTE

**Figure 1:**
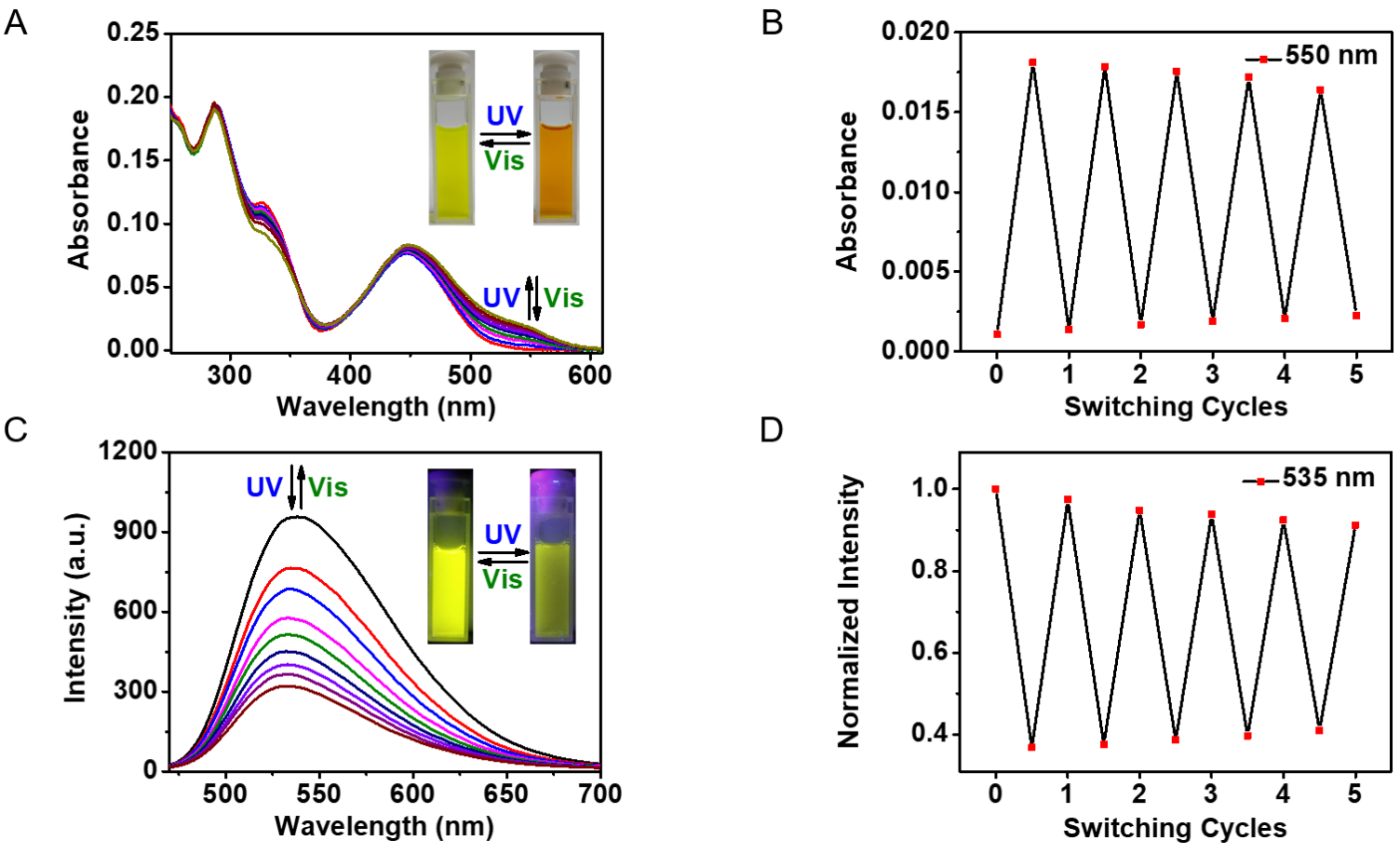
Absorption spectral changes (A), absorption fatigue resistance (B), emission spectral changes (C) and emission fatigue resistance (D) of reporter **Glyco-DTE** (1 × 10^−5^ mol/L) in PBS buffer (pH 7.4, 0.25‰ Triton X-100) upon alternating irradiation with UV (254 nm) and visible light (>500 nm). Emission spectra were produced upon excitation at 448 nm.

The photoswitching performances of **Glyco-DTE** (1 × 10^−5^ mol/L) were first measured in PBS buffer at room temperature. As shown in Figure 1A, a decreased absorption band at 327 nm and a subsequent appearance of a new band centered at 550 nm were observed upon irradiation of **Glyco-DTE** with UV light, which indicated a photocyclization or ring-closing process to form the ring-closed photoisomer [28]. The absorption band at 550 nm remained unchanged after 3 min of irradiation as the photostationary state (PSS) was reached. The absorption spectra of the ring-opened isomer could be fully recovered upon visible light irradiation (4 min), suggesting a photocycloreversion or ring-opening process from the ring-closed photoisomer to the original ring-opened photoisomer. The photo fatigue resistance of **Glyco-DTE** was then examined at 550 nm via an alternate irradiation with UV and visible light at room temperature. The ring-closing/opening cycles of **Glyco-DTE** could be repeated several times in buffer solution without obvious degradation (Figure 1B), demonstrating the robustness of **Glyco-DTE**.

Figure 1C shows the photoswitching of emission spectra of **Glyco-DTE** (1 × 10^−5^ mol/L) in PBS buffer upon alternating UV and visible light irradiation at room temperature. Upon excitation with 448 nm light, the fluorescence emission peak of **Glyco-DTE** was observed at 535 nm (Φ_F_ = 0.263, Table S1 in Supporting Information File 1). Owing to the good overlap between the emission band of the naphthalimide fluorophore and the absorption band of DTE closed isomer, the fluorescence was remarkably quenched to ca. 30% (Φ_F_ = 0.085, Table S1 in Supporting Information File 1) through an efficient intramolecular fluorescence resonance energy transfer (FRET) mechanism [29–30] after the photocyclization of **Glyco-DTE**. The fluorescence was fully recovered by irradiation with visible light and the emission fatigue resistance was also examined and found to tolerate more than five switching cycles in PBS buffer (Figure 1D). Similar performances were also observed for control reporter **8o** (Supporting Information File 1, Figure S1). The characteristic photoswitchable ON/OFF fluorescence signals as well as the robust fatigue resistance suggest the reporter designed here has great potential for photoswitchable fluorescence imaging in biological systems.

### Fabrication of Glyco-DTE@MnO_2_ glycosheets

**Figure 2:**
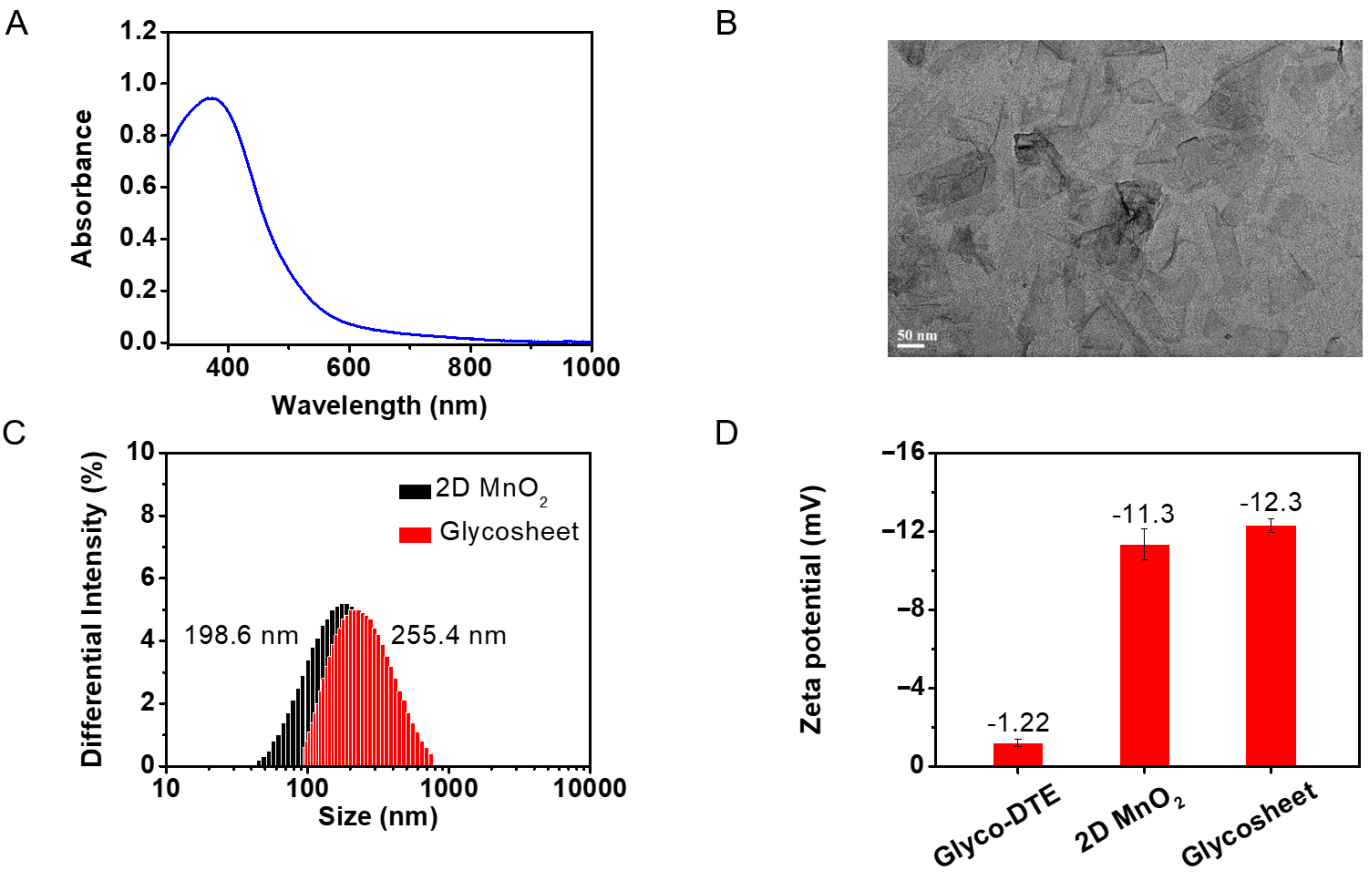
(A) The absorbance spectrum and (B) high resolution TEM image of 2D MnO_2_ nanosheets (1 × 10^−5^ g/mL) in ultrapure water; (C) DLS and Zeta potential characterizations of 2D MnO_2_ photochromic glycosheet.

The fast and simple synthesis of 2D MnO_2_ nanosheets was performed according to reported procedures [31–32], in which the freshly prepared MnO_2_ from MnCl_2_·4H_2_O was washed and sonicated in ultrapure water. As shown in Figure 2A, the obtained MnO_2_ solution exhibited a wide band in the range of 300–1000 nm with a peak located at 375 nm, which is the characteristic absorption of 2D MnO_2_ nanosheets [21,33]. The broad and strong absorption makes the 2D MnO_2_ nanosheets a potential energy acceptor for the fluorophores which are stacked on the nanosheets plane, leading to the fluorescence quenching through FRET mechanism [32,34]. The transmission electron microscopy (TEM) image of the prepared product revealed obvious morphology of nanosheets which presented a large 2D and ultrathin plane with a diameter of ca. 200 nm (Figure 2B) [33–34].

By virtue of the expansive surface, 2D MnO_2_ nanosheets possess the ability to load dozens of fluorophores. An array of fluorescent reporters thus formed, which facilitate endocytosis and significantly lower the background signal for intracellular fluorescence imaging [25–26]. Upon the incubation of the dithienylethene fluorescence reporter **Glyco-DTE** with 2D MnO_2_ nanosheets, the reporter was adsorbed on the surface of the nanosheets, forming the **Glyco-DTE@MnO_2_** photochromic glycosheet which β-D-galactosides pointing away from the surface. Dynamic light scattering (DLS) exhibited a size of 198.6 nm for the 2D MnO_2_ nanosheets (Figure 2C), which was in accordance with the TEM characterization. The size of the photochromic **Glyco-DTE@MnO_2_** glycosheet was determined as 255.4 nm, indicating the successful coating of MnO_2_ nanosheets with the **Glyco-DTE** reporter [25]. An increasing Zeta potential was also observed after the assembly, confirming again the successful fabrication of **Glyco-DTE@MnO_2_** glycosheet (Figure 2D) [26].

### GSH sensing and fluorescence photoswitching of Glyco-DTE@MnO_2_ glycosheet

**Figure 3:**
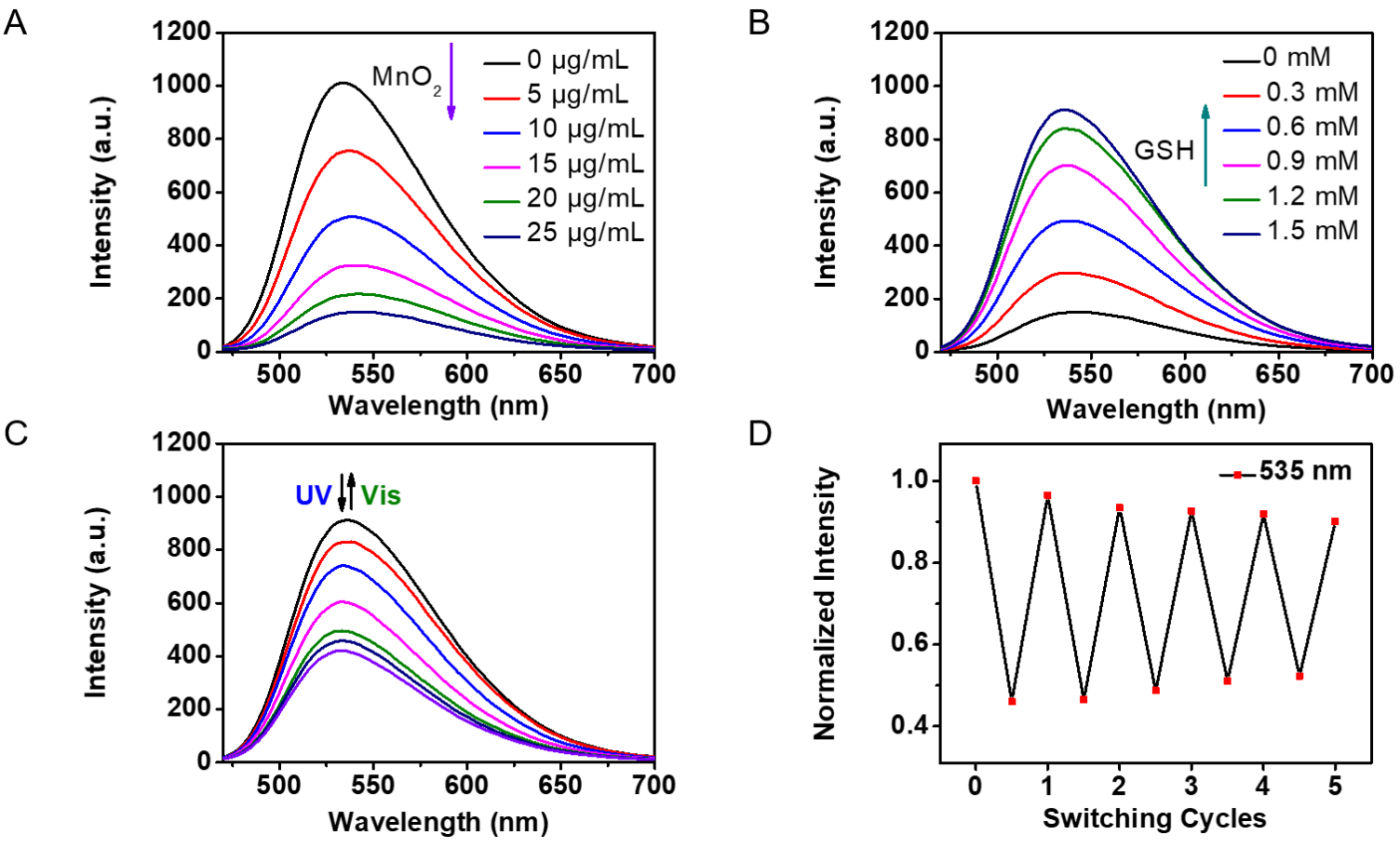
(A) Emission spectral changes of reporter **Glyco-DTE** (1 × 10^−5^ mol/L in PBS buffer, 0.25‰ Triton X-100) upon addition of 0–25 μg/mL MnO_2_, (B) emission spectral changes of glycosheet upon addition of 0–1.5 mM GSH, (C) emission spectral changes and (D) fatigue resistance of reporter **Glyco-DTE** (after the degradation of MnO_2_ with GSH) upon irradiation with UV (254 nm) and visible light (>500 nm). Emission spectra were produced upon excitation at 448 nm.

The fluorescence emission of **Glyco-DTE@MnO_2_** was efficiently quenched to ca. 15% (Φ_F_ = 0.023, Table S1 in Supporting Information File 1) when increasing concentrations of 2D MnO_2_ nanosheets were added, and reached saturation around 25 μg/mL (Figure 3A). The quenched fluorescence indicated the effective FRET between the attached **Glyco-DTE** and 2D MnO_2_, which again suggested the close stacking of **Glyco-DTE** to the nanosheet surface. Aggregation-caused quenching might be another reason for the fluorescence quenching because of the close distance between fluorescence molecules when absorbed on the surface of nanosheets. Apart from the quenched fluorescence, the photoswitchable emission was also prohibited (Figure S2, Supporting Information File 1), probably due to the significantly reduced emission operation window of **Glyco-DTE**.

We then investigated the GSH-responsive performance of **Glyco-DTE@MnO_2_** in PBS buffer. As shown in Figure 3B, the emission of **Glyco-DTE** was restored to ca. 90% (Φ_F_ = 0.256, Table S1 in Supporting Information File 1) with the addition of 1.5 mM GSH. The recovery of emission can be attributed to the reduction of MnO_2_ to Mn^2+^ [21], leading to the decomposition of MnO_2_ nanosheets. This result reveals that the photochromic glycosheet is capable of recognizing GSH, leading to a significant turn-on of the quenched fluorescence. The photoswitchable fluorescence signal was also activated alongside the recovery of naphthalimide emission. As shown in Figure 3C, the fluorescence intensity at 535 nm performed an ON/OFF switching cycle upon irradiation of UV–vis light with a decent fatigue resistance (Figure 3D), which is in good accordance with the results of **Glyco-DTE** in buffer solution.

The selectivity of **Glyco-DTE@MnO_2_** towards other intracellular species was next tested by fluorescence spectroscopy. As shown in Figure S3A (Supporting Information File 1), GSH showed a distinct selectivity over other analytes, suggesting a specific GSH detection performance of **Glyco-DTE@MnO_2_**. A linear response of the normalized fluorescent intensity *I*/*I*_max_ at 535 nm within 0–0.4 mM GSH concentration range was determined (Supporting Information File 1, Figure S3B), where *I*_max_ represents the emission intensity before the addition of MnO_2_ and *I* is the emission intensity after the addition of GSH, through which the limit of detection (LOD) was calculated to be 0.99 μM. These results demonstrate the high sensitivity of **Glyco-DTE@MnO_2_** hybrid sensor towards GSH, which allows the detection of intracellular GSH in the complex physiological environment of cells. The kinetic analysis of **Glyco-DTE@MnO_2_** in the presence of GSH (Supporting Information File 1, Figure S3C) suggests a short response time (3 min) with a reaction constant of *k* = 2.39 × 10^−2^ s^−1^ (Supporting Information File 1, Figure S3D), demonstrating a fast response of the hybrid sensor on GSH sensing.

The investigation above verifies that the photochromic glycosheet we designed can perform as an “activation and photo-switching” sensor towards GSH, which is illustrated as fluorescence turn-on and sequential on-off cycles. Besides, the quenching of the reporter fluorescence by MnO_2_ contributes to a significantly lowered background signal, which makes it an excellent material for intracellular precision imaging.

### Cell-targeted photoswitchable imaging of intracellular GSH

With the photochromic glycosheet in hand, we then investigated its applications as a biosensor for targeted intracellular GSH imaging. The presence of the β-ᴅ-galactoside residue offers a selective recognition site for ASGPr receptor which is over-expressed in HepG2 cell lines, endowing our hybrid sensor with specific cell target ability [35]. The cytoselectivity of the **Glyco-DTE** reporter was firstly checked in PBS buffer through lectin binding experiments. The lectin used here, peanut agglutinin (PNA), can selectively bind with β-ᴅ-galactoside that mimics the role of ASGPr on HepG2 cell membranes [36–37]. As shown in Figure S4 (Supporting Information File 1), the addition of PNA to the solution of **Glyco-DTE** resulted in a fluorescence enhancement with an obvious spectral blue-shift, while the addition of another lectin, conconavalin A (Con A), did not cause a substantial variation of the fluorescence spectra. For control reporter **8o**, either the addition of PNA or Con A led to minute changes in the emission spectra. The phenomena described above solidly proved the cell target ability of the β-ᴅ-galactoside moiety of the **Glyco-DTE** reporter.

**Figure 4:**
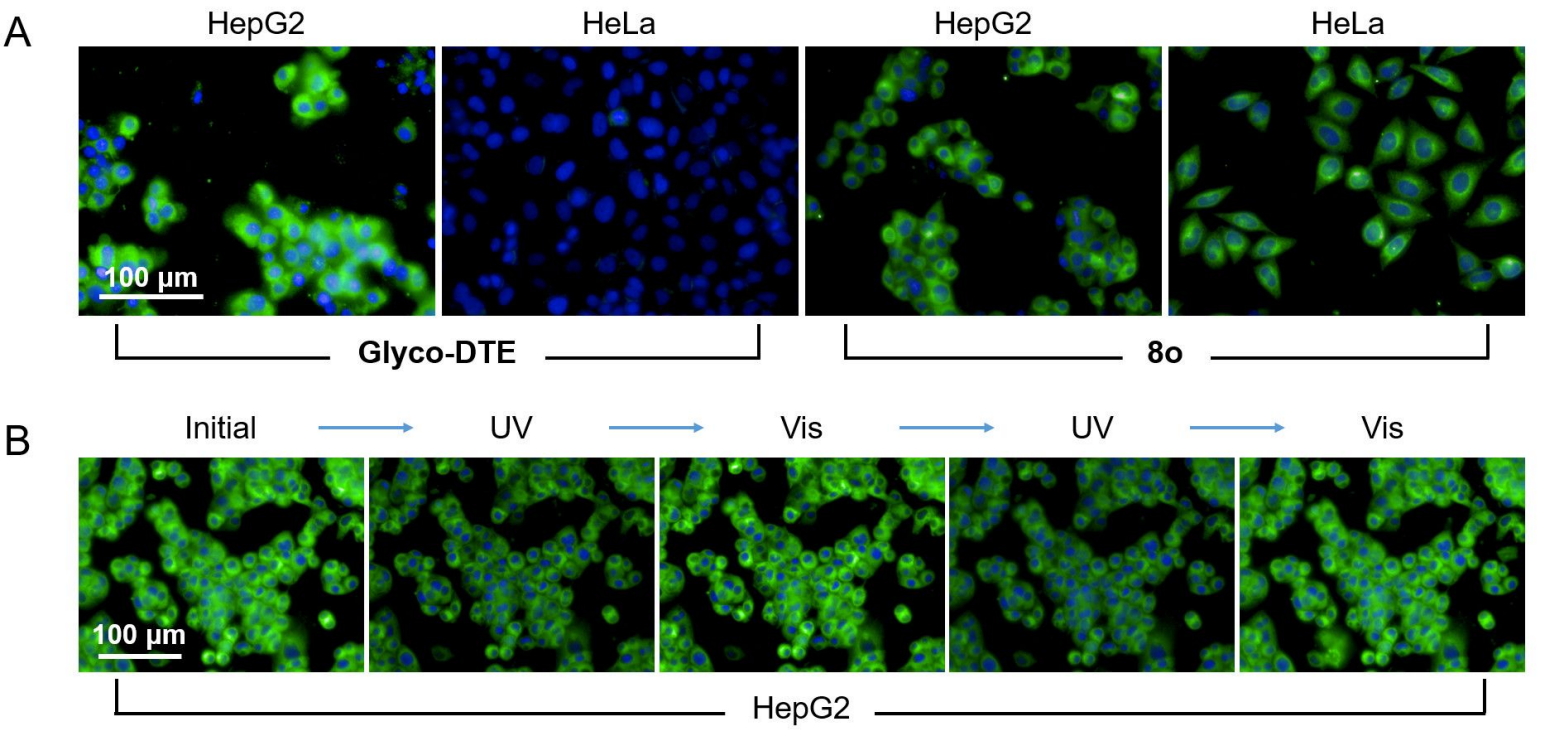
(A) Fluorescence imaging of HepG2 cells and HeLa cells after incubated with reporters **Glyco-DTE** (20 μM) and **8o** (20 μM) for 30 min. (B) The photochromic fluorescence imaging of HepG2 cells after incubated with reporter **Glyco-DTE** (20 μM) upon alternating irradiation with UV (254 nm) and visible light (>500 nm). The excitation wavelength is 440 nm and the emission channel is 450–550 nm.

In the next step, HepG2 and HeLa cells were simultaneously incubated with **Glyco-DTE** and then imaged with an Operetta high content imaging system. As shown in Figure 4A, a bright fluorescence signal was detected in HepG2 cells but almost no fluorescence signal was observed in HeLa cells. This suggested a good selectivity of **Glyco-DTE** towards HepG2 cell lines. The specific interaction between β-ᴅ-galactoside and cell transmembrane receptor ASGPr facilitates the selective cell internalization [38–39]. On the contrary, HepG2 and HeLa cells incubated with the control reporter **8o** lacking a β-ᴅ-galactoside moiety presented undiscernible fluorescence signals, confirming again the selective targeting ability of **Glyco-DTE**. Next, the intracellular photoswitchable imaging experiment of **Glyco-DTE** in HepG2 cells was operated. Upon irradiation of alternate UV–vis light, an evident fluorescence ON/OFF cycle of HepG2 cells was observed (Figure 4B). In addition to the selective internalization, **Glyco-DTE** is capable of taking remote light orders for intracellular photoswitchable imaging. The dynamic ON/OFF cycle, or photoblinking, of fluorescence from the photochromic probe guarantees the source of the signal [9]. Compared to the conventional sensor, which is vulnerable towards the inherent background signals from the intracellular environment, the photochromic probe provides a smart strategy of well-discrimination from physiological interferences.

**Figure 5:**
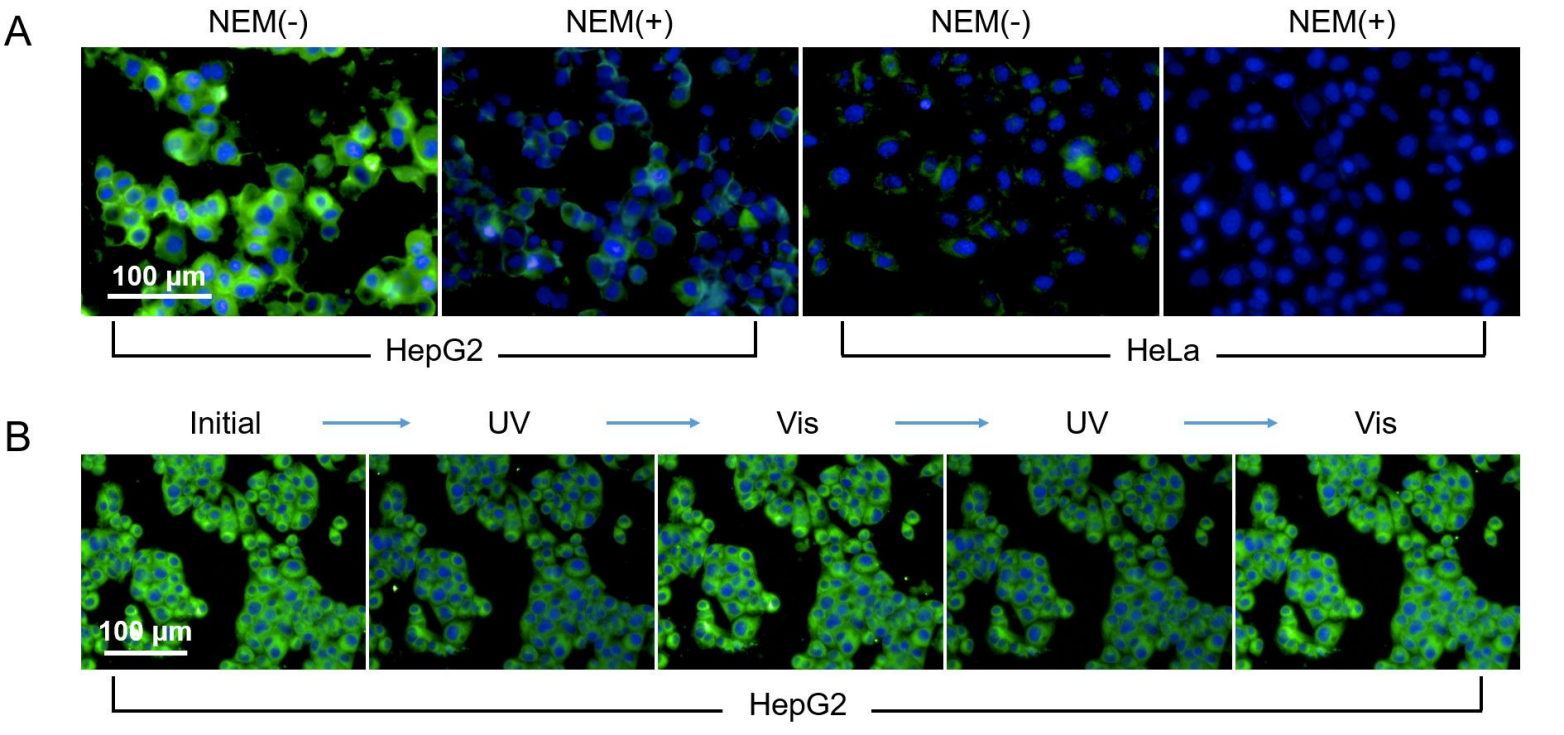
(A) Fluorescence imaging of HepG2 cells and HeLa cells after incubated with **Glyco-DTE@MnO_2_** photochromic glycosheet in the absence and presence of NEM. (B) The photochromic fluorescence imaging of HepG2 cells after incubated with glycosheet upon irradiation with UV (254 nm) and visible light (>500 nm). The excitation wavelength is 440 nm and the emission channel is 450–550 nm.

Apart from the over-expressed ASGPr receptors on cell membranes, the high intracellular concentration of GSH is another feature for HepG2 cells. Therefore, amounts of work on HepG2 cell imaging have targeted GSH as the characteristic biomarker [40–41]. Strategies like reduction of disulfide [42–44] and Michael addition [45–47] have been utilized to design fluorescent sensors for detecting intracellular GSH or discriminative sensing of GSH with other common biothiols (e.g., Cys and Hcy) [45–46]. In this work, the highly accessible 2D MnO_2_ nanosheet is used as the GSH responsive site instead of traditional functional groups that require elaborate design for high selectivity and reactivity. Besides, the incorporation of **Glyco-DTE** with MnO_2_ nanosheets quenches the fluorescence and further suppresses the background signal for intracellular imaging. To investigate the capability of our **Glyco-DTE@MnO_2_** hybrid sensor (glycosheet) for targeted photoswitchable imaging of intracellular GSH, HepG2 and HeLa cells were incubated with the glycosheet and subsequently imaged under an optical microscope. As shown in Figure 5A, HepG2 cells incubated with the glycosheet exhibited a strong fluorescence signal, indicating a high level of GSH expressed in HepG2 cells. The addition of NEM (*N*-ethylmaleimide, a GSH scavenger) resulted in a significant decrease of fluorescence intensity [40–41,46], suggesting the efficient quenching effect of MnO_2_ nanosheets towards **Glyco-DTE** reporter. In HeLa cells, as a control experiment, a fluorescence signal is hardly observed no matter treated with NEM or not. These results strongly support the feasibility of targeted intracellular GSH imaging with our **Glyco-DTE@MnO_2_** hybrid sensor. Consequently, the intracellular GSH photoswitchable imaging with the liberated **Glyco-DTE** in HepG2 cells was operated. Similar to the above results, an efficient fluorescence ON/OFF cycling upon UV–vis irradiation of HepG2 cells was performed, which provides the dynamic fluorescence signal for “double-check” of intracellular GSH. The ”first check” was the recovery of the silenced fluorescence in the presence of GSH. The following “second check” was the blinking ON/OFF fluorescence signal which ensures the origin of fluorescence signal from the photochromic probe. Hence, the intracellular sensing precision is significantly improved.

## Conclusion

In summary, photochromic glycosheet **Glyco-DTE@MnO_2_** was developed for cell-targeted and photoswitchable intracellular GSH imaging in human hepatoma cell lines. The hybrid sensing system presented here provides the MnO_2_ nanosheets for GSH detection and **Glyco-DTE** reporter for dynamic fluorescence signal modulation. Besides, the high affinity of β-ᴅ-galactoside towards ASGPr receptors on the membrane of HepG2 cells enables the specific cell-targetability of **Glyco-DTE@MnO_2_** hybrid sensor. Compared to conventional GSH biosensors, our strategy offers a simple yet smart design that circumvents the elaborate molecular design and laborious synthesis for multifunctional sensors, further broadening the availability of photochromic sensors in various physiological scenarios. These findings not only enable promising applications in targeted-cell imaging but also provide a new sensor platform useful for multiple fluorescence signaling and improving the detection precision.

## Supporting Information

File 1 Experimental procedures and spectral data.
