# Supplementary material for: Targeted photoswitchable imaging of intracellular glutathione by a photochromic glycosheet sensor
Source: Beilstein J Org Chem. 2019 Oct 7;15:2380–9. doi: 10.3762/bjoc.15.230 (PMC6808201; doi:10.3762/bjoc.15.230)
Supplement: File 1 — Experimental procedures and spectral data. [file Beilstein_J_Org_Chem-15-2380-s001.pdf]

BEILSTEIN JOURNAL OF ORGANIC CHEMISTRY

Supporting Information

for

Targeted photoswitchable imaging of intracellular glutathione by a photochromic glycosheet sensor

Xianzhi Chai, Hai-Hao Han, Yi Zang, Jia Li, Xiao-Peng He, Junji Zhang and He Tian

Beilstein J. Org. Chem. **2019,** *15,* 2380–2389. doi:10.3762/bjoc.15.230

Experimental procedures and spectral data

License and Terms: This is a supporting information file under the terms of the Creative Commons Attribution License (http://creativecommons.org/licenses/by/4.0). Please note that the reuse, redistribution and reproduction in particular requires that the authors and source are credited.
The license is subject to the *Beilstein Journal of Organic Chemistry* terms and conditions: (https://www.beilstein-journals.org/bjoc)

Table of Contents

1. Additional figures

2. Synthesis and characterization

3. Experimental procedures

4. References

5. Original spectral copies of compounds

1. Additional figures

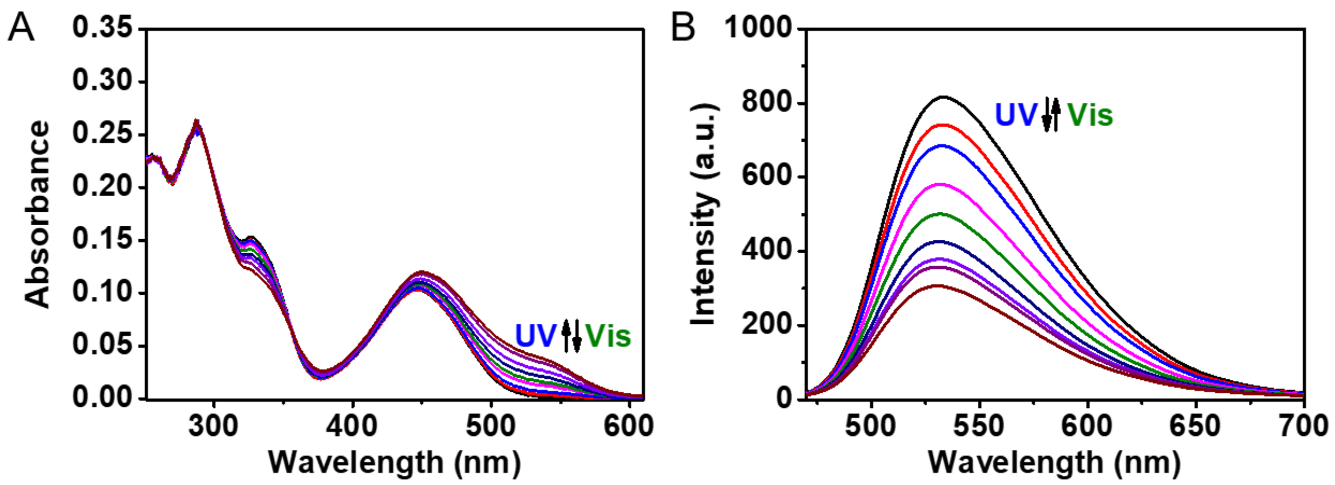

Figure S1: (A) Absorption and (B) emission spectral changes of **8o** (1×10^{-5} mol·L^{-1}) in PBS buffer (pH 7.4, 0.25‰ Triton X-100) upon irradiation with UV (254 nm) and visible light (>500 nm). Emission spectra were produced upon excitation at 448 nm.

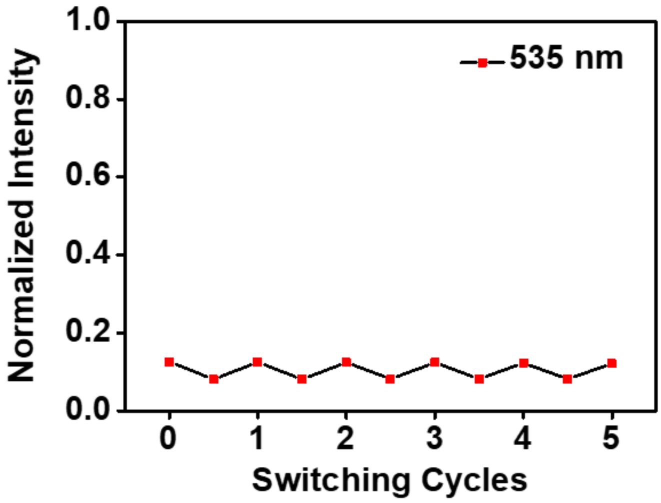

Figure S2: Fatigue resistance of **Glyco-DTE@MnO$_2$** (10 μM@25 μg/mL) in PBS buffer (pH 7.4, 0.25‰ Triton X-100) upon irradiation with UV (254 nm) and visible light (> 500 nm). Emission spectra were produced upon excitation at 448 nm.

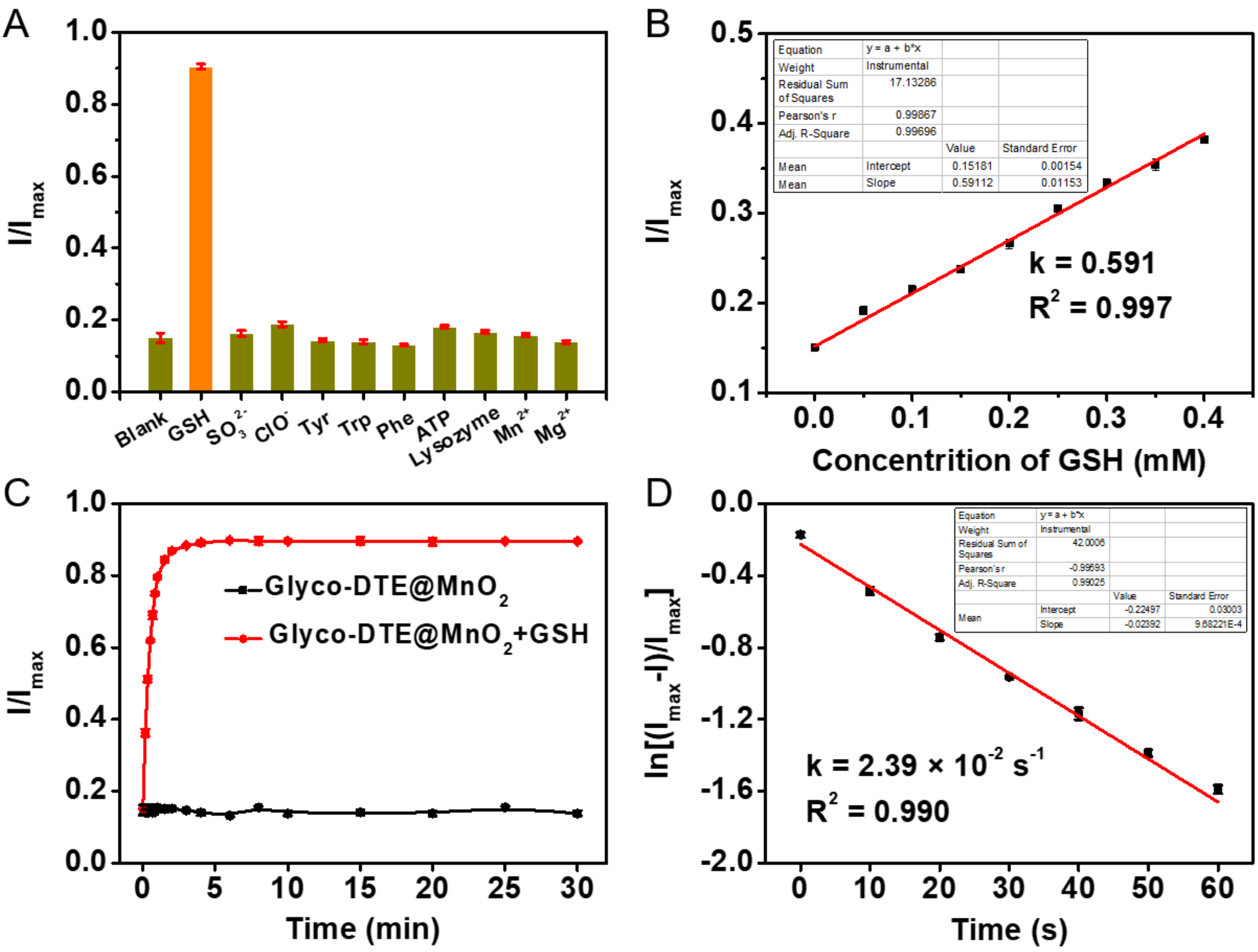

Figure S3: (A) The fluorescence responses at 535 nm of photochromic glycosheet **Glyco-DTE@MnO₂** (10 μM@25 μg/mL) toward various biomolecules (1.5 mM) or enzymes (10 U/mL) incubated for 5 min in PBS buffer (Tyr: tyrosine, Trp: tryptophan, Phe: phenylalanine, ATP: adenosine triphosphate). (B) The plot and linear fit of fluorescence intensity of **Glyco-DTE@MnO₂** at 535 nm vs. the concentration of GSH (0–0.4 mM). (C) Time dependence of the emission intensity for **Glyco-DTE@MnO₂** at 535 nm in the presence (red) and absence (black) of GSH (1.5 mM). (D) Determination of sensing kinetics: Plots of $\ln[(I_{max}-I)/I_{max}]$ as a function of time for the reaction of **Glyco-DTE@MnO₂** with GSH (1.5 mM). Emission spectra were produced upon excitation at 448 nm.

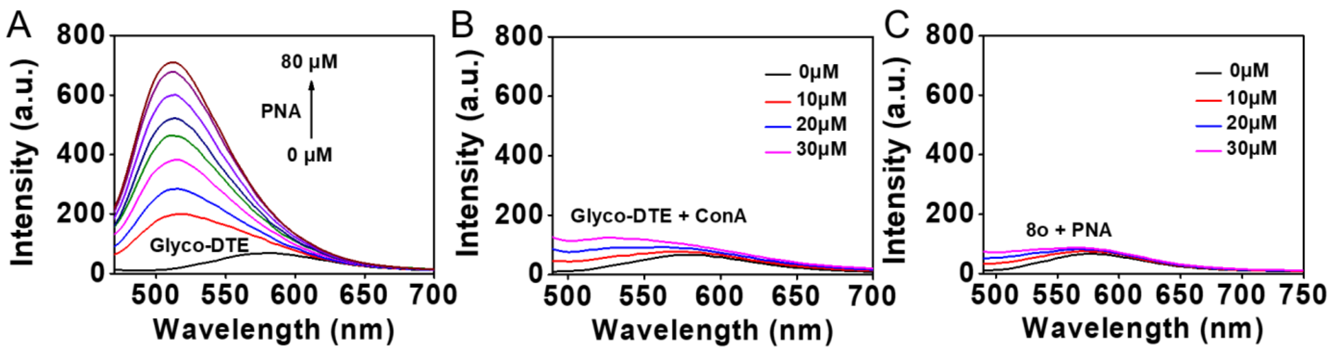

Figure S4: (A) Emission spectral changes of **Glyco-DTE** (1×10^{-6} mol/L) in PBS buffer (pH 7.4) upon addition of (A) PNA and (B) ConA. (C) Emission spectral changes of **8o** (1×10^{-6} mol/L) in PBS buffer (pH 7.4) upon addition of PNA. Emission spectra were produced upon excitation at 448 nm.

Table S1: Fluorescence quantum yields (Φ_F) of the sensor in each state

States of sensor	Φ_F
**Glyco-DTE**	0.263
**Glyco-DET** + UV	0.085
**Glyco-DTE@MnO$_2$**	0.023
**Glyco-DTE@MnO$_2$** + GSH	0.256
**Glyco-DTE@MnO$_2$** + GSH + UV	0.125

2. Synthesis and characterization

2.1 Synthesis of compound 2

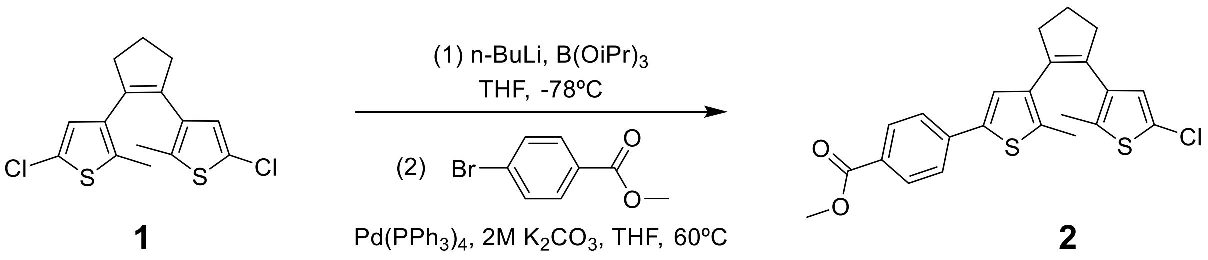

Compound **2** was synthesized according to previously reported procedures [1]. To a stirred solution of 1,2-bis(5-chloro-2-methyl-3-thienyl)cyclopentene (**1**, 1.00 g, 3.04 mmol) in THF (20 mL) at −78 °C under Ar in the absence of light, was added dropwise 2.5 M *n*-BuLi in hexane (1.50 mL, 3.00 mmol), and the reaction mixture was stirred at −70 °C for 30 min. Then, the mixture was stirred at room temperature for another 30 min and recooled to −70 °C. Then triisopropyl borate (0.85 mL, 3.04 mmol) was quickly added. The reddish solution was stirred overnight at room temperature, and used in the Suzuki cross coupling reaction without further purification.

A mixture of methyl 4-bromobenzoate (0.65 g, 3.04 mmol), the catalyst Pd $(PPh_3)_4$ (702 mg, 0.61 mmol) and THF (10 mL) was stirred for 15 min at room temperature. Then aqueous K_2CO_3 (8 mL, 2 M) was added. The resulting mixture was heated to 60 °C and the solution of the above-mentioned boric acid ester was added dropwise by syringe. Subsequently, the mixture was heated for 6 h at 60 °C and then cooled to room temperature. The resulting mixture was poured into H_2O and extracted with dichloromethane. The organic layer was collected and the solvent removed under reduced pressure to produce a brown solid. The solid was further purified by silica gel column chromatography using PE/DCM 2:1 (v/v) as eluent to obtain compound **2** as pale brown liquid (0.54 g, yield 42%). ^{1}H NMR (400 MHz, $CDCl_3$) δ 8.00 (d, J = 8.5 Hz, 2H), 7.54 (d, J = 8.5 Hz, 2H), 7.10 (s, 1H), 6.61 (s, 1H), 3.91 (s, 3H), 2.85 – 2.71 (m, 4H), 2.09 – 2.03 (m, 2H), 2.00 (s, 3H), 1.88 (s, 3H). HRMS-ESI (m/z): $[M + Na]^+$ calcd. for $C_{23}H_{21}ClO_2S_2Na^+$ 451.0463, found 451.0568.

2.2 Synthesis of compound 3

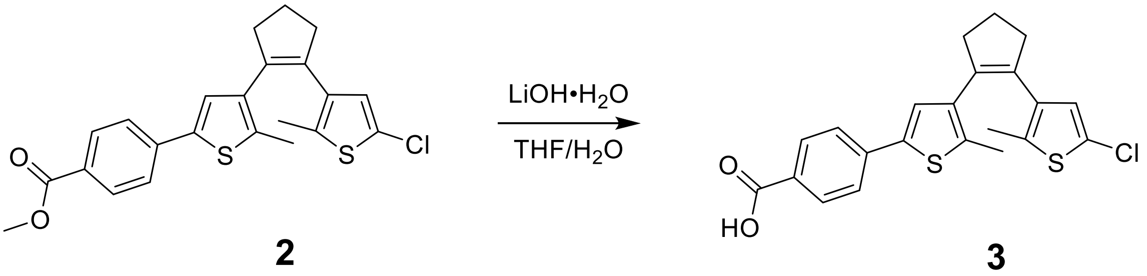

In a 50 mL two-neck round-bottomed flask, the solution of compound **2** (1.28 g, 3 mmol) in THF (15 mL) was stirred at 0 °C in an ice–water bath. Then, $LiOH{\cdot}H_2O$ (1.26 g, 30 mmol) in H_2O (6 mL) was added slowly. The reaction was monitored ceaselessly by TLC until compound **2**

completely disappeared. Afterwards, 30 mL DCM were added and the suspension was filtered to retain the filtrate. The combined organic phase was concentrated under reduced pressure and the residue was purified by silica gel column chromatography using DCM/MeOH 50:1 (v/v) as eluent to obtain compound **3** as white solid (0.91 g, yield 72%). ^{1}H NMR (400 MHz, $CDCl_3$) δ 8.08 (d, J = 8.5 Hz, 2H), 7.57 (d, J = 8.5 Hz, 2H), 7.13 (s, 1H), 6.62 (s, 1H), 2.82 (t, J = 7.4 Hz, 2H), 2.75 (t, J = 7.4 Hz, 2H), 2.10 – 2.04 (m, 2H), 2.01 (s, 3H). ^{13}C NMR (101 MHz, $CDCl_3$) δ 171.93, 139.52, 138.39, 136.93, 136.67, 135.02, 134.92, 134.31, 133.30, 130.91, 127.35, 126.78, 125.67, 125.20, 124.91, 38.47, 38.40, 22.94, 14.58, 14.24. HRMS-ESI (m/z): $[M - H]^-$ calcd. for $C_{22}H_{18}ClO_2S_2^-$ 413.0437, found 413.0436.

2.3 Synthesis of compound 4

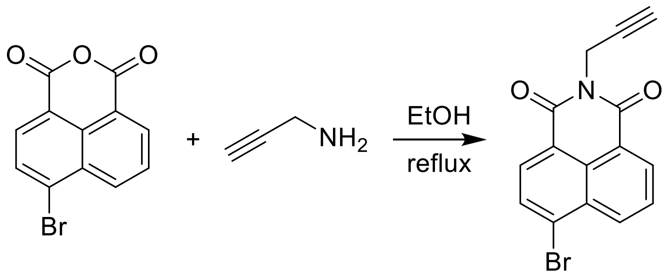

4

To a stirred solution of 4-bromo-1,8-naphthalic anhydride (1.11 g, 4 mmol) in EtOH (20 mL) was added 2-propynylamine (0.22 g, 4 mmol) and the mixture was heated for 4 hours under reflux and Ar. After cooling to room temperature, the suspension was filtered to retain the residue. The residue was washed with EtOH three times and then dried in vacuum to give compound **4** as offwhite powder (0.93 g, yield 74%). ^{1}H NMR (400 MHz, $CDCl_3$) δ 8.69 (dd, J = 7.3, 0.9 Hz, 1H), 8.58 (dd, J = 8.5, 1.0 Hz, 1H), 8.44 (d, J = 7.9 Hz, 1H), 8.05 (d, J = 7.9 Hz, 1H), 7.86 (dd, J = 8.4, 7.4 Hz, 1H), 4.95 (d, J = 2.5 Hz, 2H), 2.21 (t, J = 2.5 Hz, 1H). ^{13}C NMR (101 MHz, $CDCl_3$) δ 162.85, 133.74, 132.47, 131.62, 131.21, 130.82, 130.72, 129.02, 128.18, 122.75, 121.87, 78.33, 70.75, 29.56. HRMS-ESI (m/z): $[M + H]^+$ calcd. for $C_{15}H_9BrNO_2^+$ 313.9817 found 313.9812.

2.4 Synthesis of compound 5

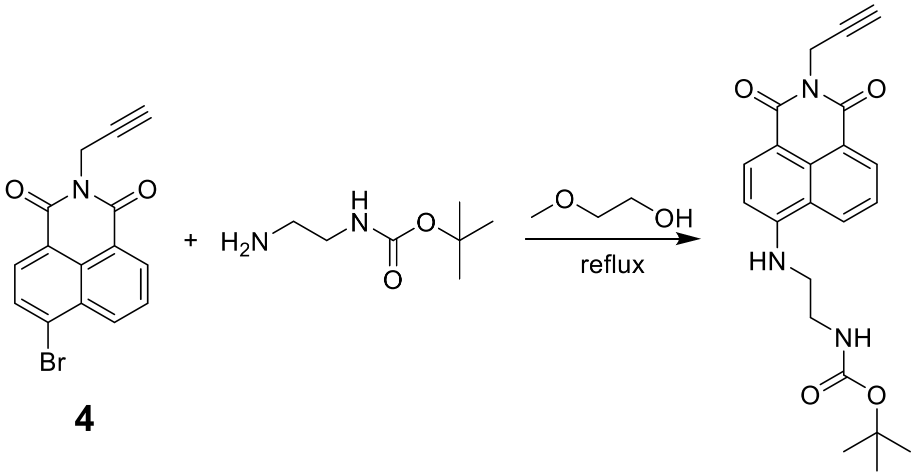

4

5

To a 50 mL two-neck round-bottomed flask, compound **4** (1.26 g, 4 mmol), *N*-Boc-1,2-ethylenediamine (0.64 g, 4 mmol) and methoxyethanol (10 mL) were added. The mixture was heated to 110 °C for 4 h under Ar. After cooling to room temperature, 30 mL H_2O were poured in and the residue filtered. The crude was purified by silica gel column chromatography using DCM as eluent to obtain compound **5** as yellow solid (0.85 g, yield 54%). ^1H NMR (400 MHz, DMSO-d_6) δ 8.59 (d, J = 8.4 Hz, 1H), 8.42 (d, J = 7.2 Hz, 1H), 8.23 (d, J = 6.4 Hz, 1H), 7.80 (s, 1H), 7.67 (t, J = 6.8 Hz, 1H), 7.11 (t, J = 5.7 Hz, 1H), 6.80 (d, J = 6.9 Hz, 1H), 4.74 (d, J = 2.0 Hz, 1H), 3.44 (d, J = 5.7 Hz, 1H), 3.29 (d, J = 6.0 Hz, 1H), 3.10 (t, J = 2.4 Hz, 1H), 1.39 (s, 5H). ^{13}C NMR (101 MHz, DMSO-d_6) δ 162.95, 161.92, 156.00, 150.88, 134.35, 130.86, 129.31, 128.74, 124.23, 121.36, 120.01, 107.18, 103.69, 79.90, 77.93, 72.46, 42.94, 28.60, 28.17. HRMS-ESI (m/z): $[M+H]^+$ Calcd. for $C_{26}H_{24}N_3O_4^+$ 394.1767, found 394.1769.

2.5 Synthesis of compound 6

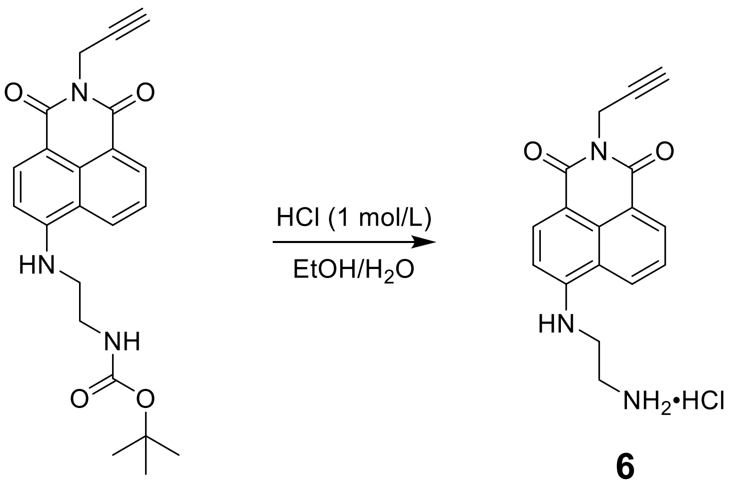

6

5

To a 50 mL two-neck round-bottomed flask, compound **5** (1.0 g, 2.54 mmol) in EtOH (11 mL) was added followed by the slow addition of 12 M HCl (1 mL) under cooling in an ice–water bath. The resulting mixture was stirred for 5 hours at room temperature. The residue was

filtered and dried in vacuum to obtain compound **6** as yellow solid (0.75 g, yield 90%). ^{1}H NMR (400 MHz, DMSO-d$_6$) δ 8.84 (d, J = 8.3 Hz, 1H), 8.39 (d, J = 7.1 Hz, 1H), 8.35 (s, 3H), 8.22 (d, J = 8.5 Hz, 1H), 8.07 (s, 1H), 7.69 – 7.61 (m, 1H), 6.81 (d, J = 8.7 Hz, 1H), 4.71 (d, J = 2.2 Hz, 2H), 3.68 (d, J = 5.2 Hz, 2H), 3.18 (dd, J = 11.1, 5.4 Hz, 2H), 3.11 (t, J = 2.4 Hz, 1H). ^{13}C NMR (101 MHz, DMSO-d$_6$) δ 162.93, 161.97, 150.46, 134.27, 130.93, 129.60, 129.16, 124.34, 121.24, 120.28, 107.86, 103.99, 79.86, 72.55, 40.39, 37.24, 28.65.

2.6 Synthesis of compound 7

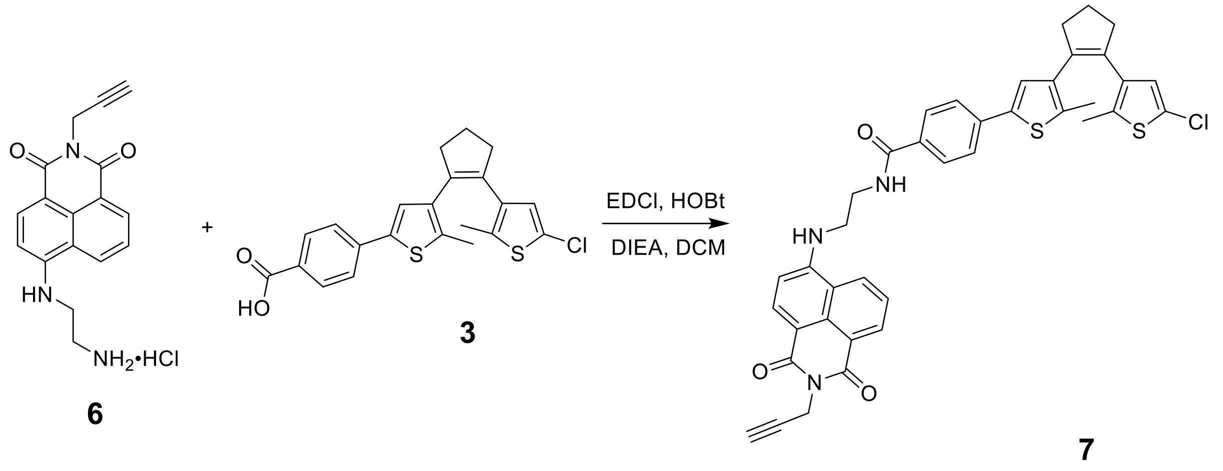

Compound **3** (1.00 g, 2.41 mmol), 1-(3-dimethylaminopropyl)-3-ethylcarbodiimide hydrochloride (EDCI, 0.92 g, 4.83 mmol) and 1-hydroxybenzotriazole (HOBt, 0.98 g, 7.24 mmol) were dissolved in 20 mL anhydrous DCM under stirring at 0 °C in an ice–water bath for 30 min. Then compound **6** (0.79 g, 2.41 mmol) in anhydrous DCM and *N*,*N*-diisopropylethylamine (DIEA, 1.99 mL, 12.06 mmol) was added and the mixture stirred at room temperature overnight. The solution was washed with 10 mL saturated salt solution for three times and the solvent removed under reduced pressure. The residue was purified by silica gel column chromatography using DCM as eluent to obtain compound **7** as yellow solid (1.04 g, yield: 62.3%). ^{1}H NMR (400 MHz, DMSO-d$_6$) δ 8.80 (s, 1H), 8.57 (d, J = 8.5 Hz, 1H), 8.38 (d, J = 7.3 Hz, 1H), 8.21 (d, J = 8.5 Hz, 1H), 7.96 (s, 1H), 7.90 (d, J = 8.3 Hz, 2H), 7.63 (t, J = 7.5 Hz, 3H), 7.37 (s, 1H), 6.85 (d, J = 8.7 Hz, 1H), 6.81 (s, 1H), 4.72 (s, 2H), 3.71 – 3.51 (m, 4H), 3.08 (t, J = 2.2 Hz, 1H), 2.75 (dt, J = 22.3, 6.7 Hz, 4H), 1.98 (dt, J = 17.5, 8.6 Hz, 2H), 1.89 (s, 3H), 1.83 (s, 3H). ^{13}C NMR (101 MHz, DMSO-d$_6$) δ 166.30, 162.93, 161.91, 150.79, 137.99, 136.60, 136.20, 135.13, 134.85, 134.71, 134.31, 133.46, 132.93, 132.46, 130.80, 129.29, 128.66, 128.03, 127.23, 125.41, 124.41, 124.25, 123.56, 121.35, 120.02, 107.25, 103.73, 79.90, 72.43, 42.60, 37.97, 37.79, 28.61, 22.22, 13.97, 13.77. HRMS-ESI (m/z): $[M + H]^+$ calcd. for $C_{39}H_{33}ClN_3O_3S_2^+$ 690.1652, found 690.1660.

2.7 Synthesis of compound 8o

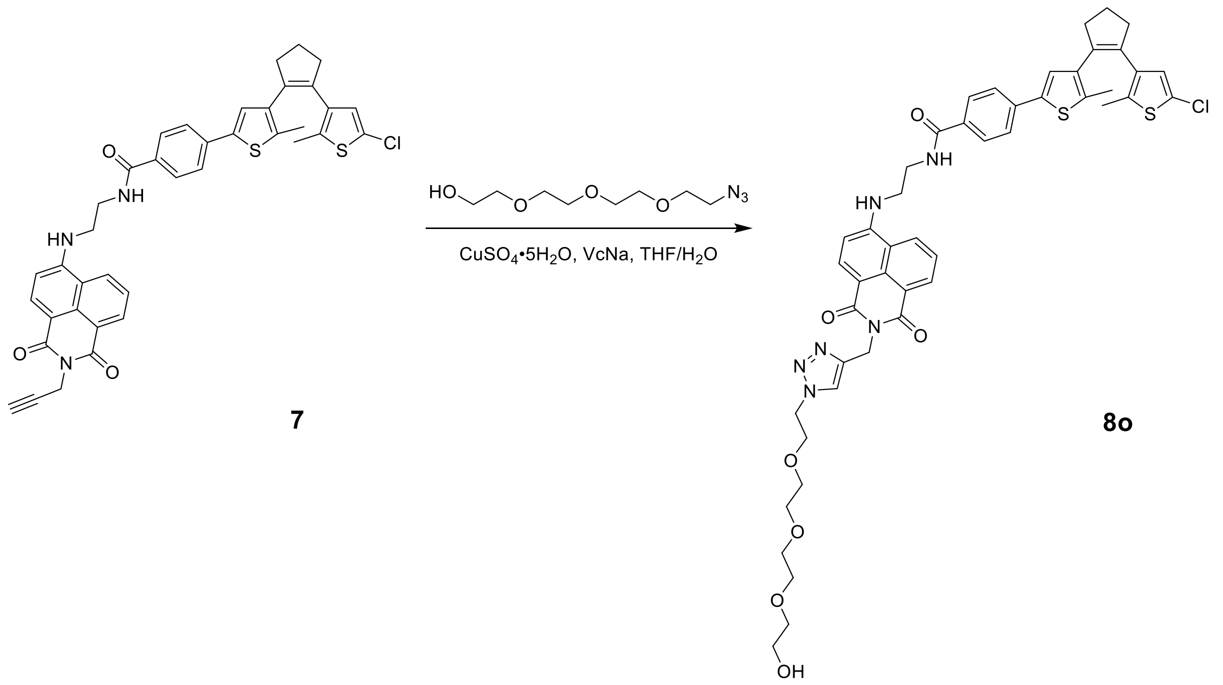

To a 50 mL two-neck round-bottomed flask, compound **7** (0.69 g, 1 mmol), mono-azide-substituted tetraglycol (0.22 g, 1 mmol), $CuSO_4·5H_2O$ (0.5 g, 2 mmol) and THF (10 mL) were added. Then, sodium ascorbate (VcNa, 0.80 g, 4 mmol) was slowly added and the mixture was stirred under Ar at room temperature overnight. Afterwards, 30 mL DCM were added and the combined organic phase was concentrated under reduced pressure. The residue was purified by silica gel column chromatography using DCM/MeOH 20:1 (v/v) as eluent to obtain compound **8o** as yellow solid (0.81 g, yield 89%). ^{1}H NMR (400 MHz, $CDCl_3$) δ 8.28 (s, 1H), 7.99 (d, J = 8.2 Hz, 1H), 7.92 (d, J = 8.7 Hz, 3H), 7.80 (d, J = 7.1 Hz, 1H), 7.69 (d, J = 8.1 Hz, 1H), 7.49 (d, J = 8.2 Hz, 2H), 7.05 (s, 1H), 6.84 (t, J = 7.6 Hz, 1H), 6.59 (s, 1H), 6.27 (d, J = 8.3 Hz, 1H), 4.53 (s, 2H), 4.06 (s, 3H), 3.90 (s, 2H), 3.85 (s, 2H), 3.75 (s, 2H), 3.66 – 3.45 (m, 13H), 2.76 (dt, J = 14.7, 6.5 Hz, 4H), 2.08 – 2.01 (m, 2H), 1.98 (s, 3H), 1.87 (s, 3H). ^{13}C NMR (101 MHz, $CDCl_3$) δ 168.88, 164.38, 163.61, 151.01, 143.98, 138.47, 137.44, 136.75, 135.93, 135.05, 134.97, 134.52, 134.16, 133.25, 132.25, 130.42, 129.06, 128.14, 127.47, 126.80, 125.04, 124.85, 123.93, 121.15, 120.00, 108.12, 103.49, 72.47, 70.51, 70.35, 70.18, 69.29, 61.62, 50.51, 44.85, 39.14, 38.44, 38.34, 34.48, 22.90, 14.49, 14.23. HRMS-ESI (m/z): $[M + Na]^+$ calcd. for $C_{47}H_{49}ClN_6O_7S_2Na^+$ 931.2690, found 931.2688.

2.8 Synthesis of compound 9

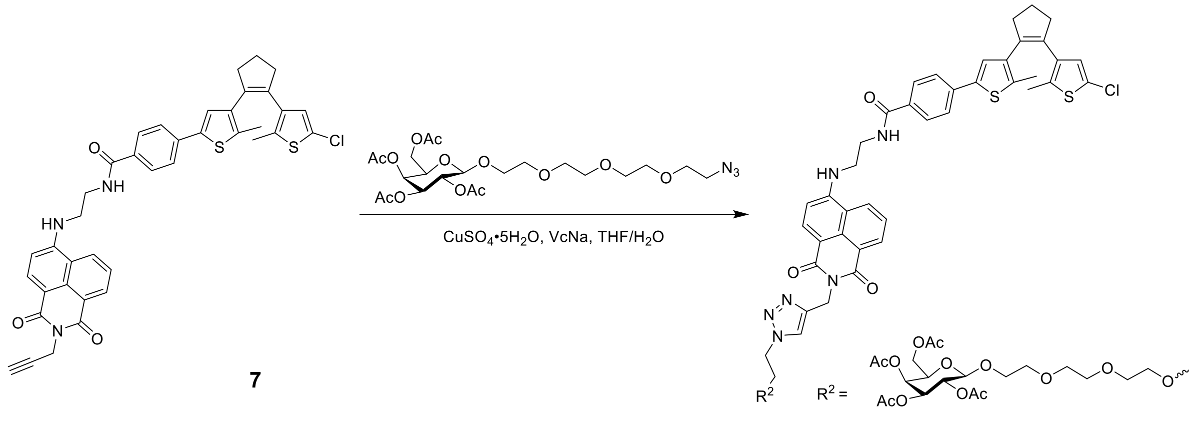

To a 50 mL two-neck round-bottomed flask, compound **7** (0.69 g, 1 mmol), glycosyl-substituted tetraglycol (0.55 g, 1 mmol), $CuSO_4·5H_2O$ (0.5 g, 2 mmol) and THF (10 mL) were added. Then, VcNa (0.80 g, 4 mmol) was slowly added and the mixture was stirred under Ar at room temperature overnight. Afterwards, 30 mL DCM were added and the combined organic phase was concentrated under reduced pressure. The residue was purified by silica gel column chromatography using DCM/MeOH 20:1 (v/v) as eluent to obtain compound **9** as yellow solid (1.06 g, yield 86%). ^{1}H NMR (400 MHz, $CDCl_3$) δ 8.17 (s, 1H), 8.05 (s, 1H), 7.99 – 7.86 (m, 4H), 7.82 (s, 1H), 7.50 (d, J = 7.8 Hz, 2H), 7.43 (s, 1H), 7.05 (s, 1H), 6.93 (s, 1H), 6.60 (s, 1H), 6.31 (s, 1H), 5.44 – 5.29 (m, 3H), 5.23 – 5.17 (m, 1H), 5.03 (dd, J = 10.4, 2.9 Hz, 1H), 4.60 – 4.49 (m, 3H), 4.20 – 4.10 (m, 2H), 3.93 (dd, J = 25.0, 18.2 Hz, 5H), 3.73 (dd, J = 10.7, 4.3 Hz, 1H), 3.68 – 3.47 (m, 13H), 2.77 (dd, J = 12.7, 7.4 Hz, 4H), 2.14 (s, 4H), 2.10 – 1.91 (m, 17H), 1.88 (s, 3H). ^{13}C NMR (101 MHz, $CDCl_3$) δ 170.44, 170.30, 170.19, 169.55, 168.70, 151.07, 138.50, 137.40, 136.74, 135.89, 135.05, 134.99, 134.16, 133.25, 132.36, 128.11, 126.80, 125.05, 124.87, 101.35, 70.92, 70.65, 70.57, 70.44, 70.23, 69.09, 68.84, 67.08, 61.28, 38.43, 38.33, 29.69, 22.90, 20.80, 20.69, 20.60, 14.48, 14.22. HRMS-ESI (m/z): $[M + H]^+$ calcd. for $C_{61}H_{68}ClN_6O_{16}S_2^+$ 1239.3822, found 1239.3864.

2.9 Synthesis of compound Glyco-DTE

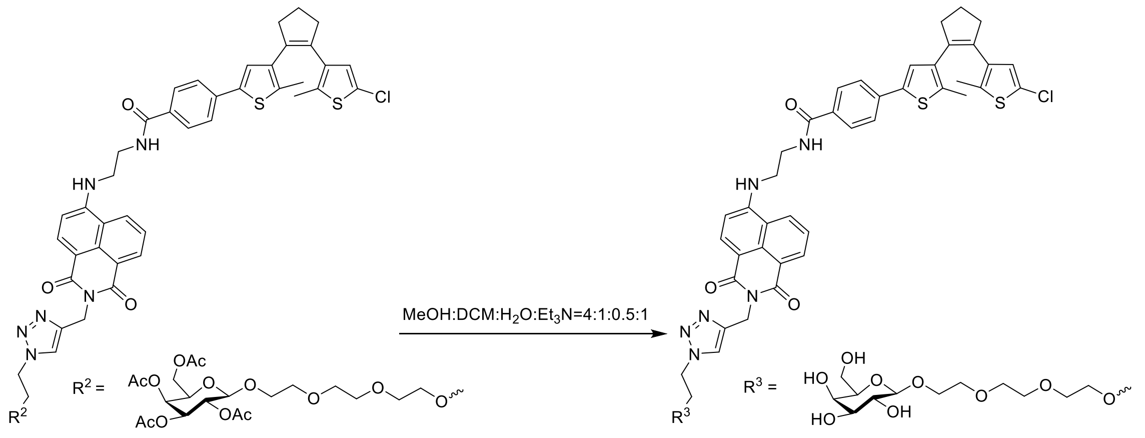

9 **Glyco-DTE**

To a stirring solution of compound **9** (1.24 g, 1 mmol) in DCM (8 mL) was added MeOH (2 mL), H_2O (1 mL) and trimethylamine (2 mL). The resulting mixture was left for 3 hours at room temperature. The mixture was concentrated under reduced pressure and the residue was purified by silica gel column chromatography using DCM/MeOH 10:1 (v/v) as eluent to obtain compound **Glyco-DTE** as yellow solid (0.86 g, yield 80%). ^{1}H NMR (400 MHz, $CDCl_3$) δ 8.83 (s, 1H), 8.68 (d, J = 8.4 Hz, 1H), 8.44 (d, J = 7.2 Hz, 1H), 8.28 (d, J = 8.5 Hz, 1H), 8.02 (s, 1H), 7.92 (s, 1H), 7.89 (d, J = 7.8 Hz, 2H), 7.70 (t, J = 7.7 Hz, 1H), 7.63 (d, J = 8.3 Hz, 2H), 7.40 (s, 1H), 6.94 (d, J = 8.6 Hz, 1H), 6.85 (s, 1H), 5.26 (s, 2H), 4.84 (d, J = 4.2 Hz, 1H), 4.70 (d, J = 4.8 Hz, 1H), 4.58 (t, J = 5.5 Hz, 1H), 4.44 (t, J = 5.1 Hz, 2H), 4.36 (d, J = 4.5 Hz, 1H), 4.08 (d, J = 6.9 Hz, 1H), 3.84 – 3.78 (m, 1H), 3.76 (t, J = 5.2 Hz, 2H), 3.60 (s, 5H), 3.56 – 3.43 (m, 9H), 3.35 – 3.21 (m, 5H), 3.17 (d, J = 5.0 Hz, 1H), 3.06 (q, J = 7.3 Hz, 1H), 2.85 – 2.69 (m, 4H), 2.00 (dt, J = 14.1, 7.0 Hz, 2H), 1.91 (s, 3H), 1.85 (s, 3H). ^{13}C NMR (101 MHz, DMSO-d_6) δ 166.27, 163.51, 162.58, 150.77, 143.32, 137.99, 136.64, 136.18, 135.16, 134.86, 134.75, 134.35, 133.50, 132.96, 132.48, 130.88, 129.50, 128.67, 128.04, 127.28, 125.46, 124.43, 123.54, 121.75, 120.16, 107.64, 103.81, 103.53, 75.15, 73.42, 70.45, 69.65, 69.55, 69.44, 68.60, 68.10, 67.65, 60.38, 49.22, 31.25, 29.78, 28.98, 28.66, 22.21, 22.06, 13.99, 13.91, 13.79, 8.57. HRMS-ESI (m/z): $[M + Na]^+$ calcd. for $C_{53}H_{59}ClN_6O_{12}S_{12}Na^+$ 1093.3219, found 1093.3214.

3. Experimental procedures

Materials and general methods

All solvents and chemicals were purchased from commercial suppliers in analytical grade and used without further purification unless otherwise indicated. Peanut agglutinin (PNA) and concanavalin A (ConA) were supplied by Sigma. $MnCl_2 \cdot 4H_2O$, H_2O_2 and tetramethylammonium hydroxide were purchased from Admas. Absorption and emission spectra were recorded using a Varian Cary 500 and Varian Cary Eclipse, respectively. The UV (365 nm, 160 mW cm^{-2}) and light-emitting diode (LED) lamps (>500 nm, 150 mW cm^{-2}) were used as light sources for UV and visible-light irradiation, respectively. DLS and Zeta-potential measurement were performed with a Zetasizer Nano. High-resolution transmission electron microscopy (HRTEM) analysis was performed with a JEM-2100. Cell fluorescence imaging experiments were performed with an Operetta high content imaging system (Perkin Elmer, USA).

Preparation of MnO_2 nanosheets

The MnO_2 nanosheets were synthesized according to a reported procedure [2]. $MnCl_2 \cdot 4H_2O$ (724 mg, 3.65 mmol) was dissolved in a small amount of ultrapure water, and then a mixture of 1 mol/L tetramethylammonium hydroxide (18 mL) and 30 wt % H_2O_2 (3 mL) were added to the above solution within 15 s. The formed dark-brown suspension was stirred vigorously overnight open to the air at room temperature, which was accompanied by the generation of oxygen. Then, the obtained dark brown solution was centrifuged at 4000 rpm for 10 min to collect the precipitate, which was washed with water and methanol several times. Next, the precipitate was dissolved in water and sonicated for 10 hours. The solution was centrifuged at 4000 rpm for 10 min to collect the supernatant, which was lyophilized and finally dissolved in water with a concentration of 5 mg/mL.

Photochromic tests in solution

The compound was dissolved in DMSO first and then diluted to 10 μM in PBS buffer (pH 7.4) with 0.25‰ Triton X-100 (v/v). The compound solution was irradiated with UV light (365 nm, 160 mW cm^{-2}) and visible light (>500 nm, 150 mW cm^{-2}) alternately in a darkroom. After every irradiation for a suitable time, the absorption and emission spectral changes of the compound were measured until the curve didn't change anymore.

Cell culture

HepG2 and Hela cells were cultured at 37 °C under a humidified 5% CO_2 atmosphere in Dulbecco's Modified Eagle's Medium (Invitrogen, Carlsbad, CA, USA) supplemented with 10% fetal bovine serum (Gibco, Gland Island, NY, USA).

Fluorescence imaging and photochromic fluorescence imaging of cells

Cells were cultured in growth medium supplemented with 10% FBS. Then, cells (2.0 × 10^4/well) were seeded on a black 96-well microplate with optically clear bottom (Greiner bio-one, Germany) overnight, and then incubated with 20 μM of sensor for 30 min. After three rinses in

PBS, the fluorescence was detected and photographed with an Operetta high content imaging system.

For the photochromic fluorescence imaging measurement, the cells were pretreated with 4% paraformaldehyde and incubated with probes. To test the intracellular photochromic cycling, the 96-well microplate was irradiated with UV light (365 nm, 160 mW cm^{-2}) in a darkroom for 10 min, and fluorescence detected and photographed with the Operetta high content imaging system. Subsequently, the 96-well microplate was reversibly irradiated with vis light (>500 nm, 150 mW cm^{-2}) in the darkroom for 10 min, and fluorescence detected and photographed with the Operetta high content imaging system.

4. References

[1] Lin, Q.; Xiao, S.; Li, R.; Tan, R.; Wang, S.; Zhang, R. *Dyes Pigm.* **2015**, *114*, 33-39.
[2] Yuan, Y.; Wu, S.; Shu, F.; Liu, Z. *Chem. Commun.* **2014**, *50*, 1095-1097.

5. Original spectral copies of new compounds

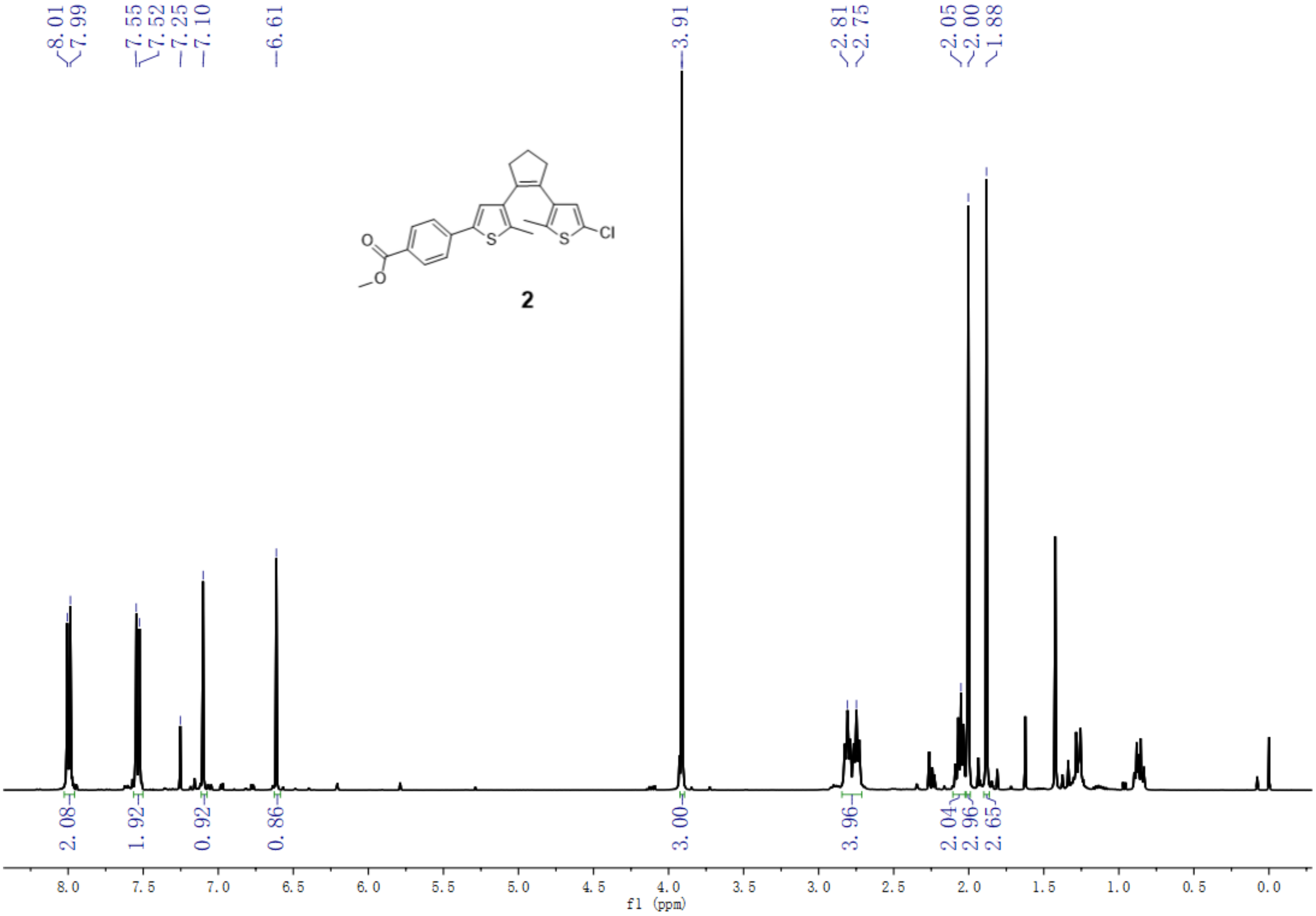

^{1}H NMR spectrum of compound **2**

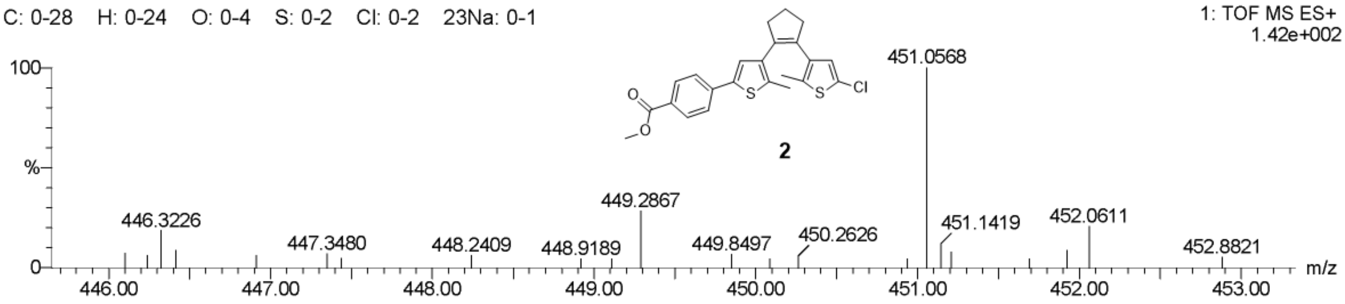

Minimum:				-1.5			
Maximum:		30.0	50.0	100.0			
Mass	Calc. Mass	mDa	PPM	DBE	i-FIT	i-FIT (Norm)	Formula
451.0568	451.0463	10.5	23.3	20.5	40.3	0.0	C27 H15 O3 S2

HRMS spectrum of compound **2**

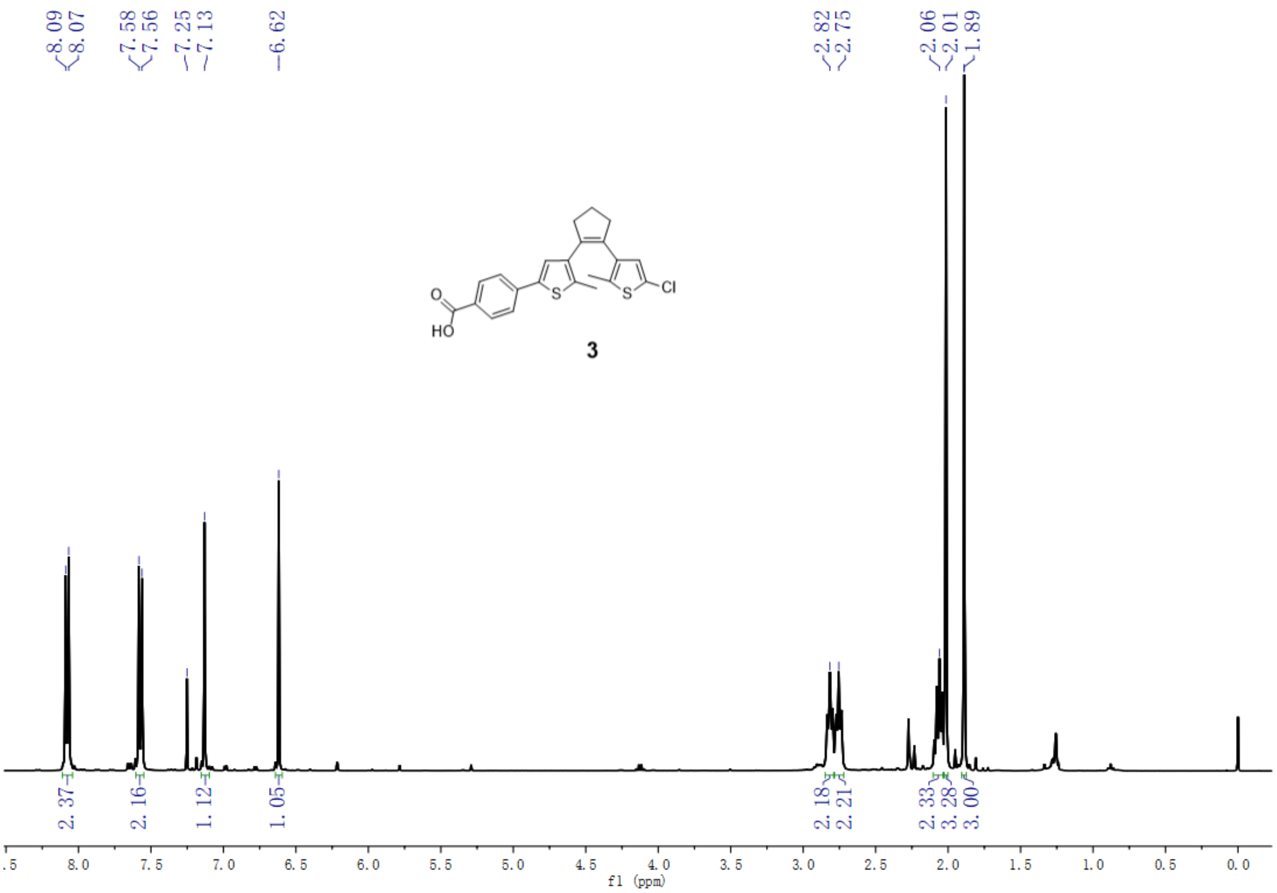

^{1}H NMR spectrum of compound **3**

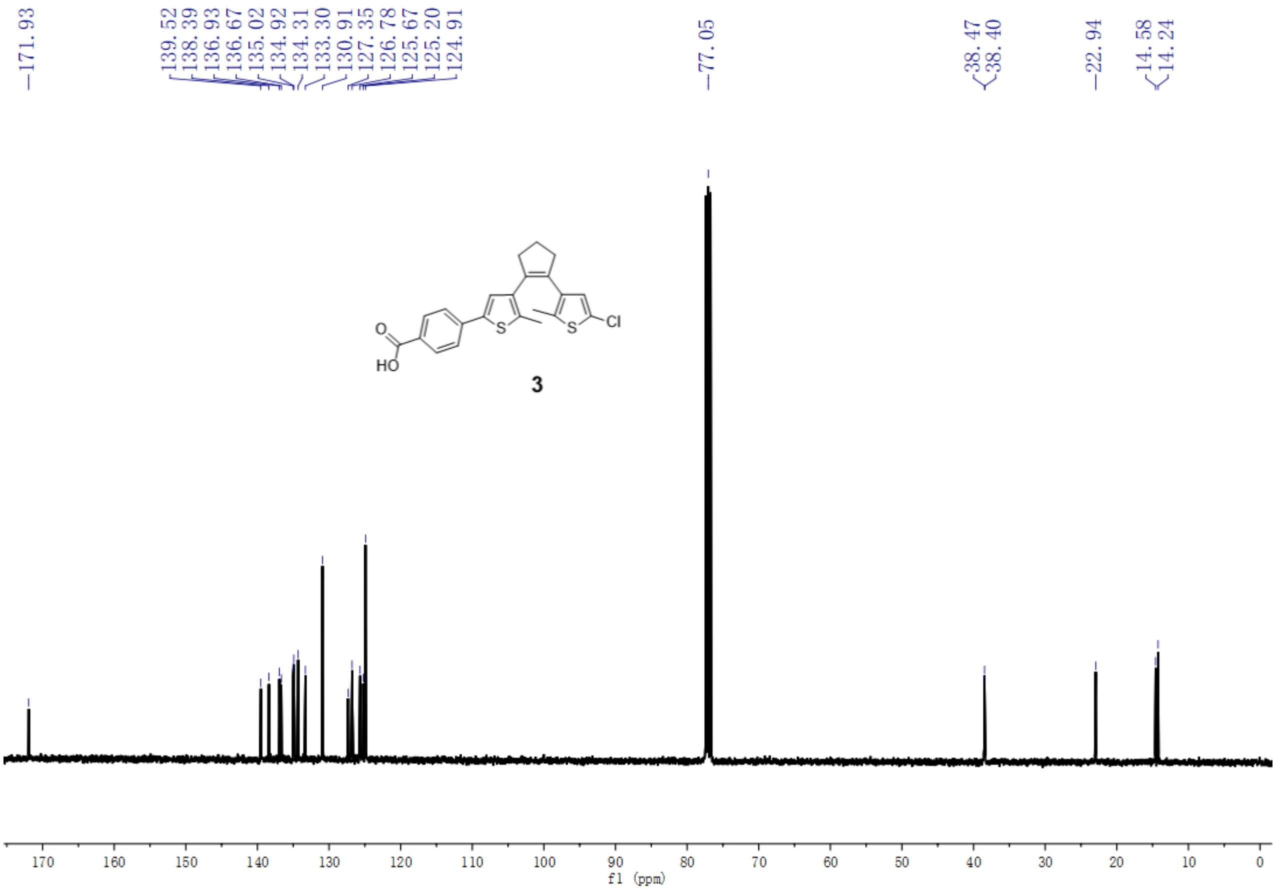

^{13}C NMR spectrum of compound **3**

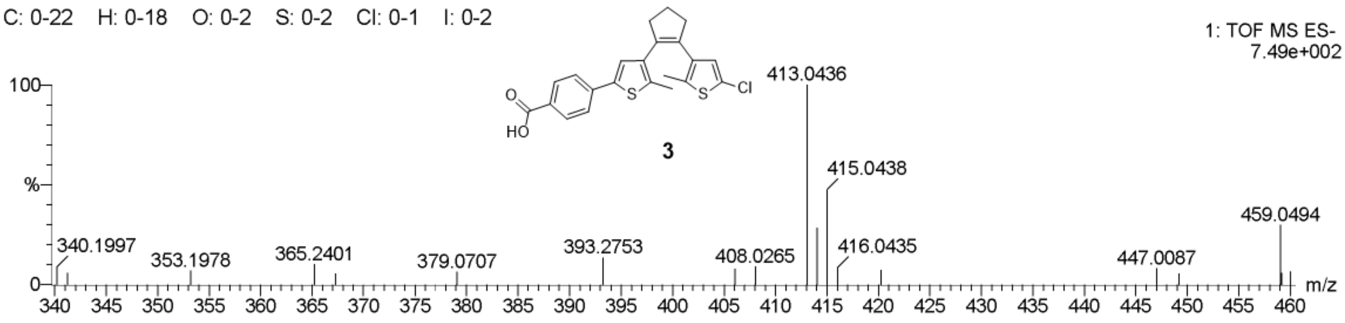

Minimum:				-1.5			
Maximum:		30.0	50.0	100.0			
Mass	Calc. Mass	mDa	PPM	DBE	i-FIT	i-FIT (Norm)	Formula
413.0436	413.0437	-0.1	-0.2	13.5	7.0	0.0	C22 H18 O2 S2 Cl

HRMS spectrum of compound **3**

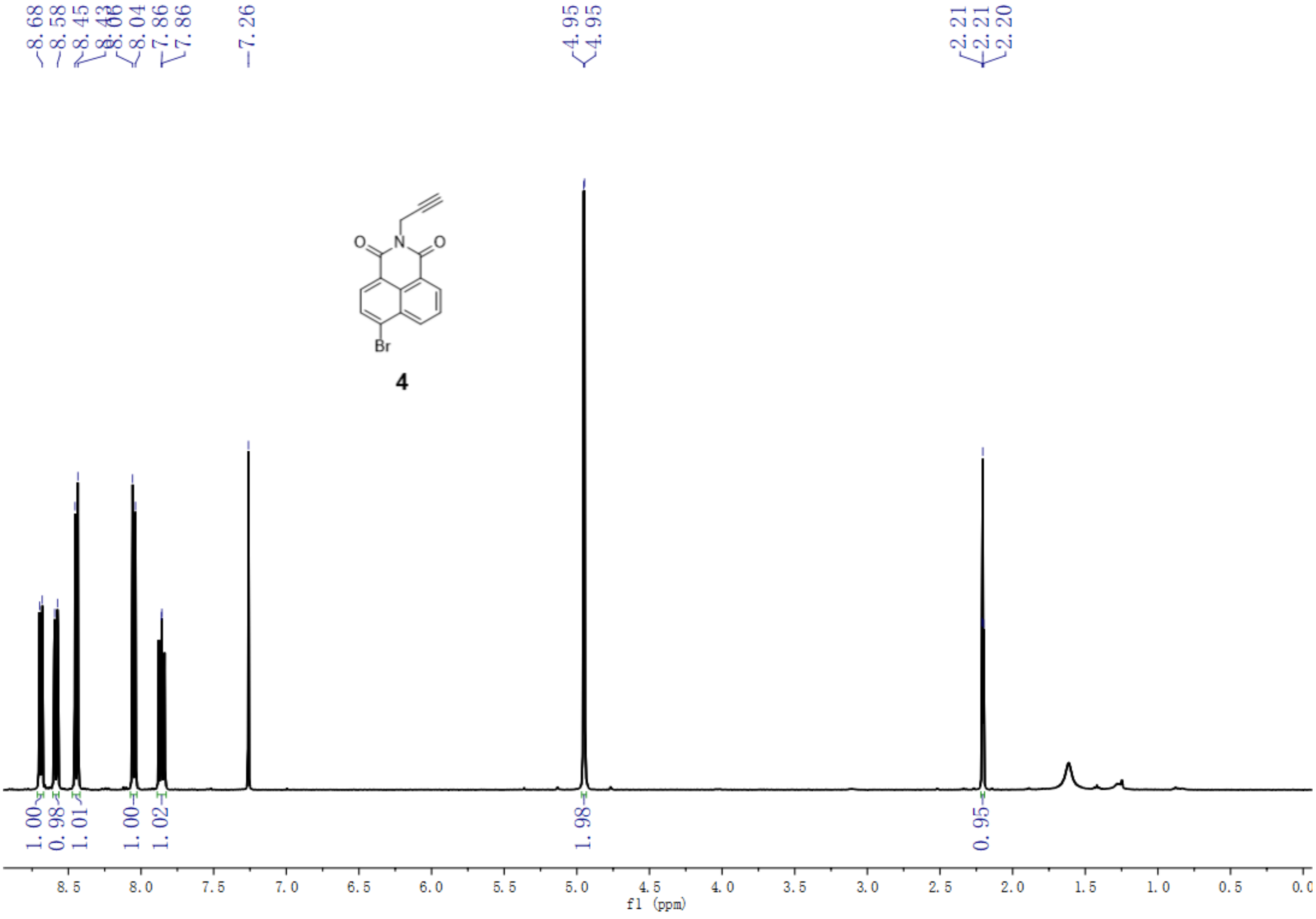

^{1}H NMR spectrum of compound **4**

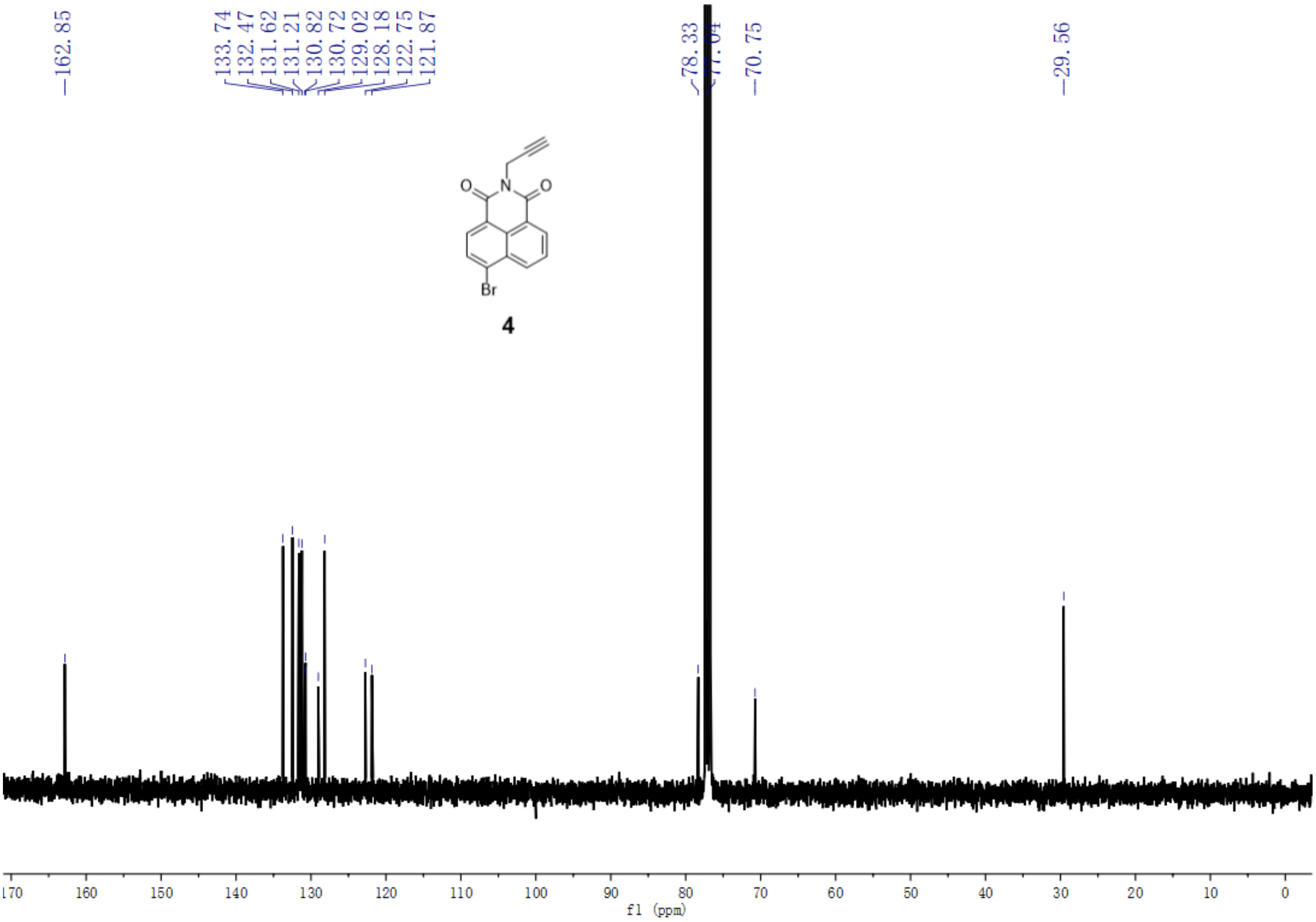

^{13}C NMR spectrum of compound **4**

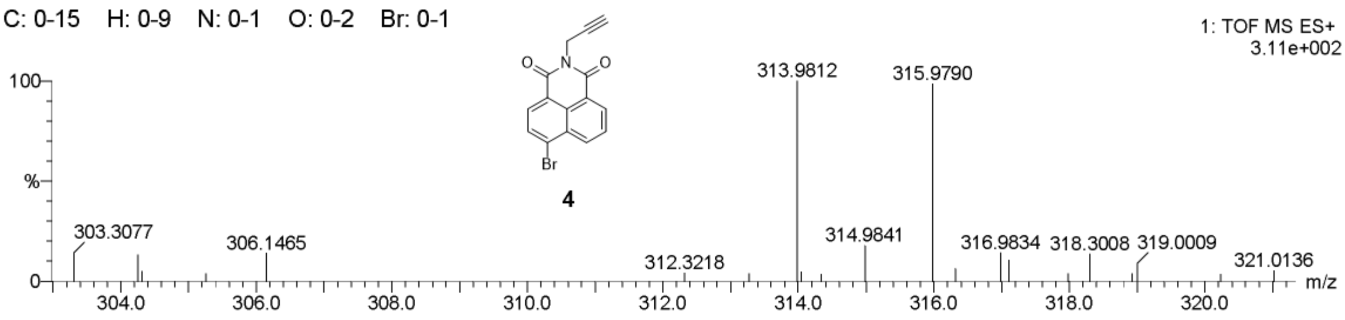

Minimum:				-1.5			
Maximum:		30.0	50.0	100.0			
Mass	Calc. Mass	mDa	PPM	DBE	i-FIT	i-FIT (Norm)	Formula
313.9812	313.9817	-0.5	-1.6	11.5	20.1	0.0	C15 H9 N O2 Br

HRMS spectrum of compound **4**

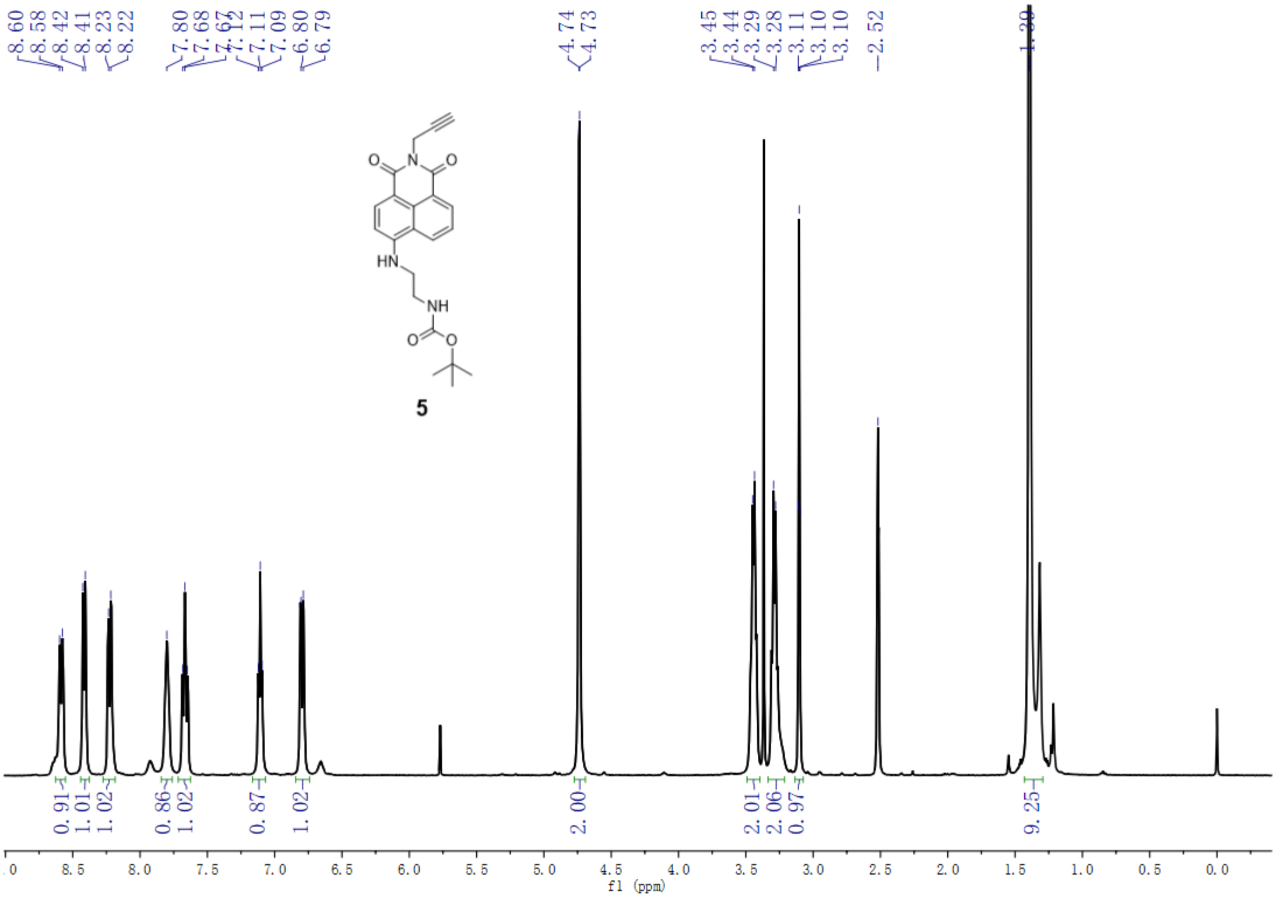

^{1}H NMR spectrum of compound **5**

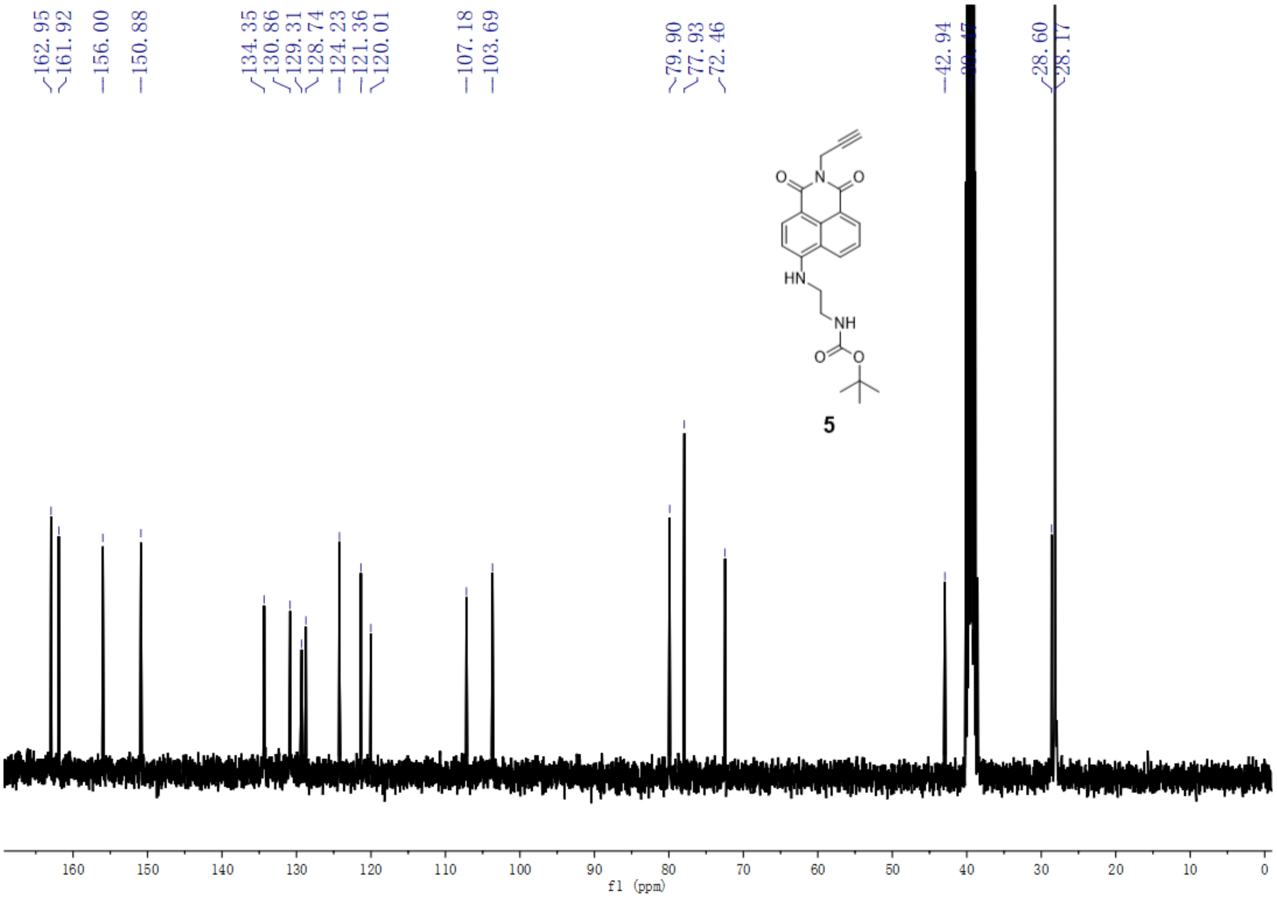

^{13}C NMR spectrum of compound **5**

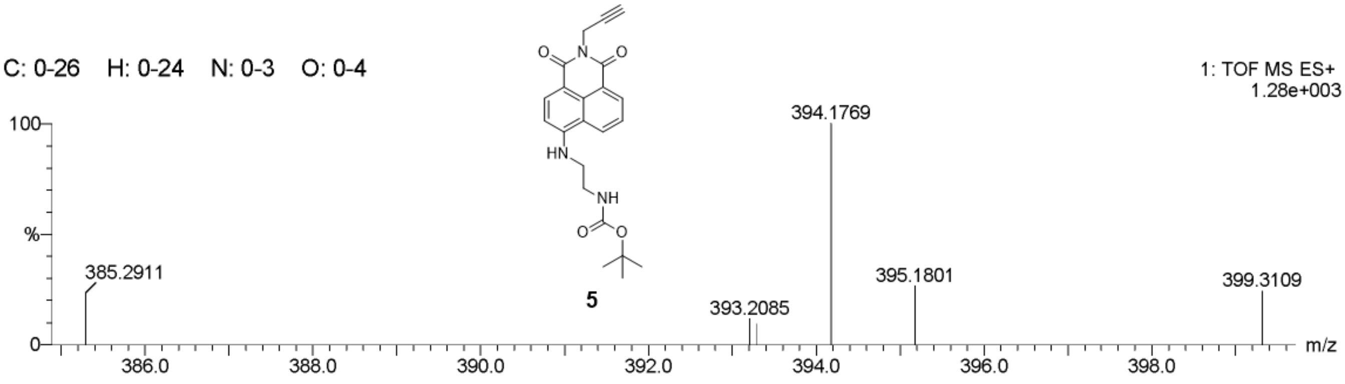

Minimum:				-1.5
Maximum:		5.0	20.0	50.0

Mass	Calc. Mass	mDa	PPM	DBE	i-FIT	i-FIT (Norm)	Formula
394.1769	394.1767	0.2	0.5	12.5	6.3	0.0	C22 H24 N3 O4

HRMS spectrum of compound **5**

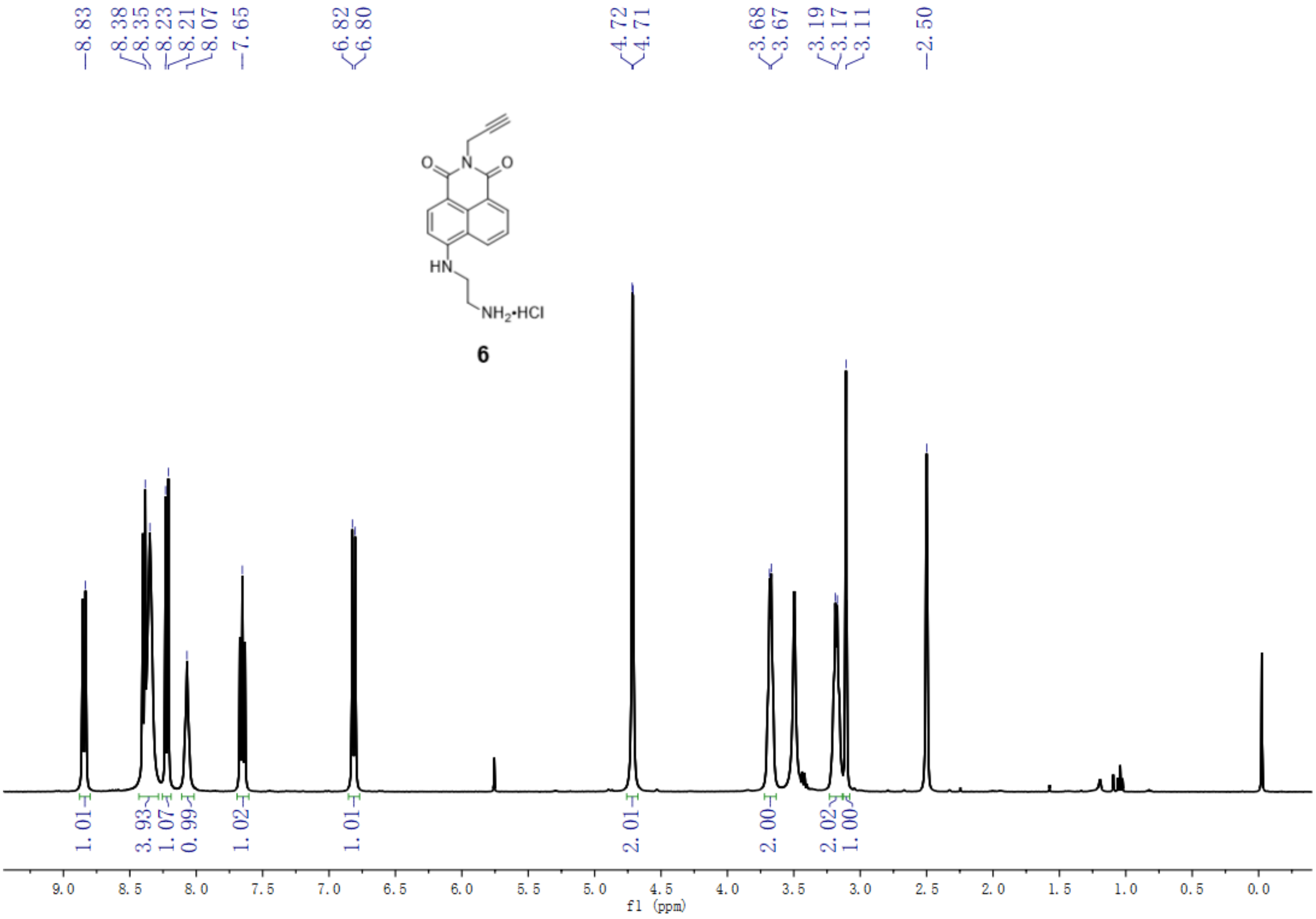

^{1}H NMR spectrum of compound **6**

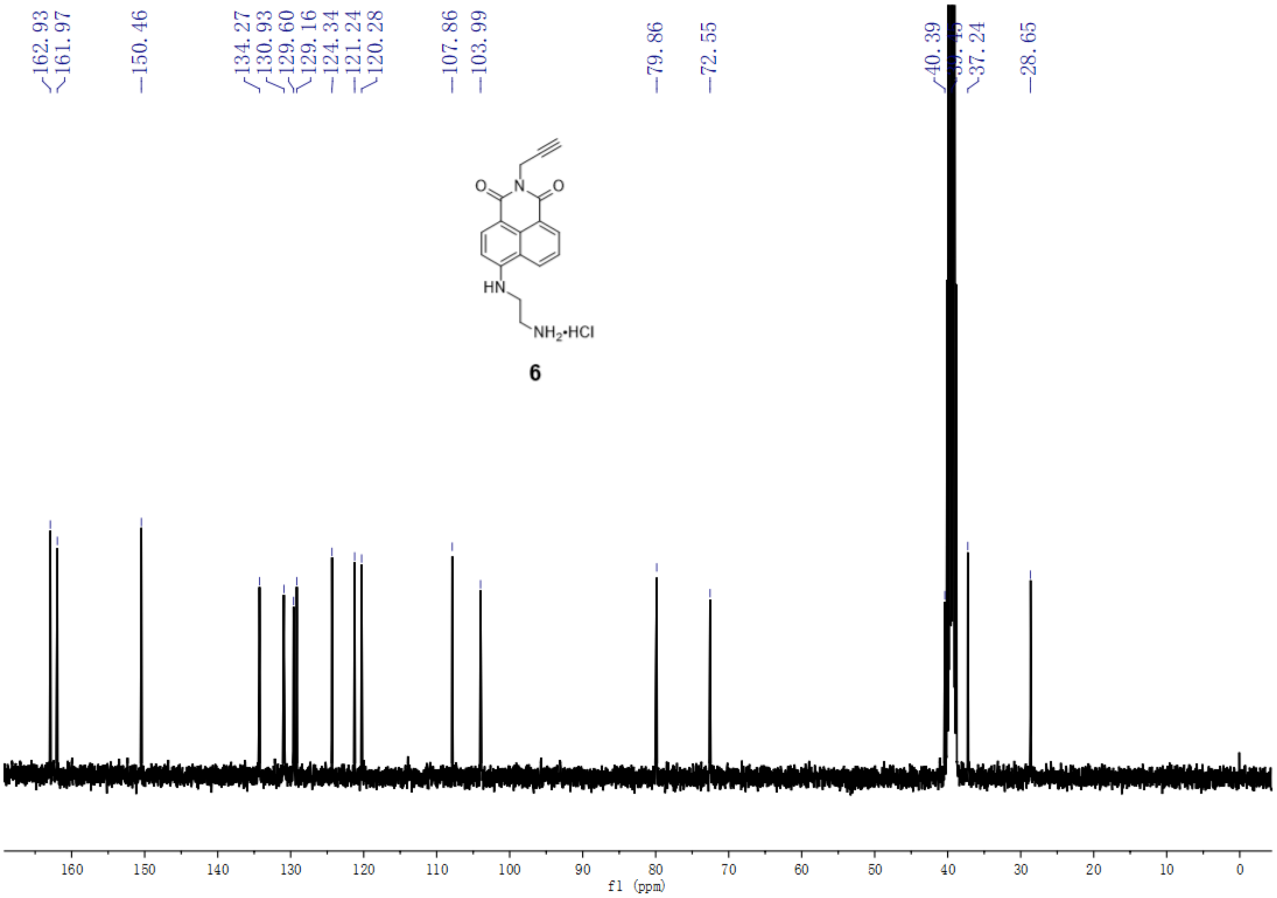

^{13}C NMR spectrum of compound **6**

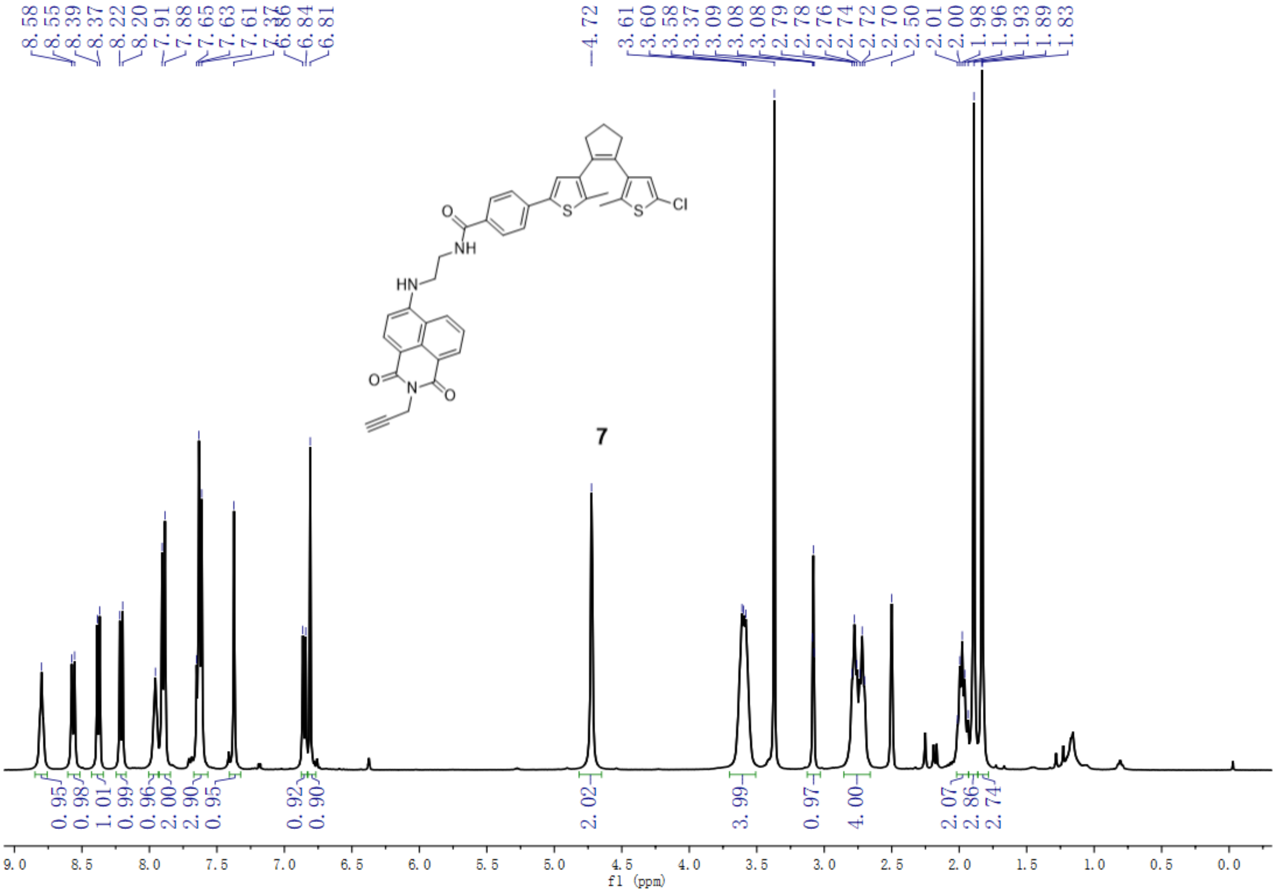

^{1}H NMR spectrum of compound **7**

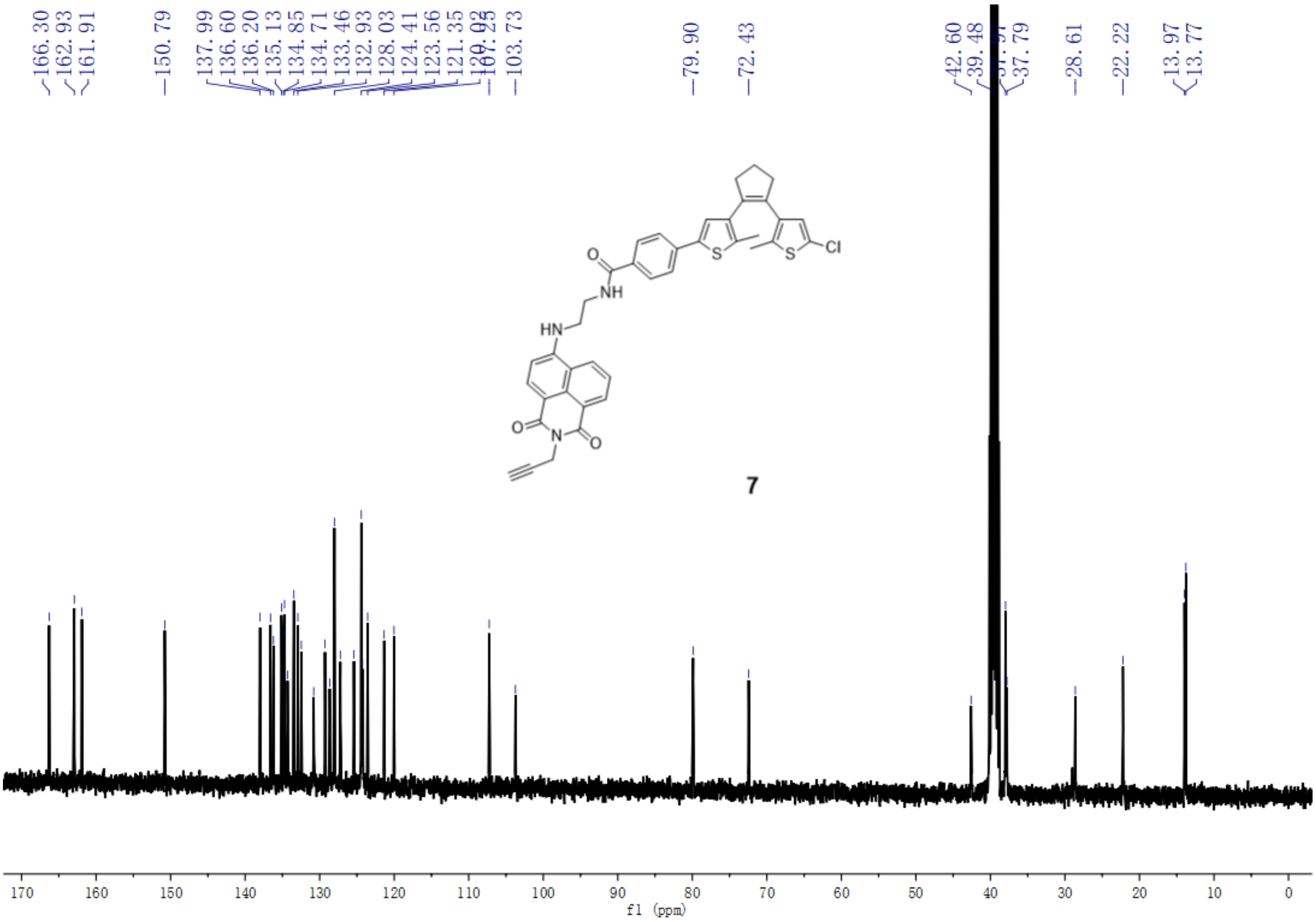

^{13}C NMR spectrum of compound **7**

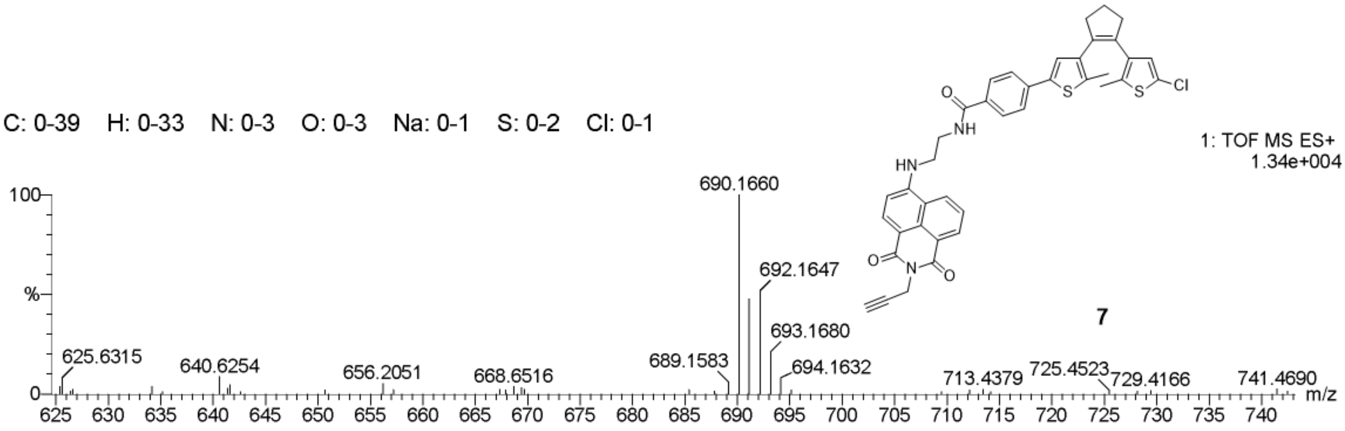

Minimum:				-1.5			
Maximum:		30.0	50.0	100.0			
Mass	Calc. Mass	mDa	PPM	DBE	i-FIT	i-FIT (Norm)	Formula
690.1660	690.1652	0.8	1.2	24.5	9.0	0.0	C39 H33 N3 O3 S2 Cl

HRMS spectrum of compound **7**

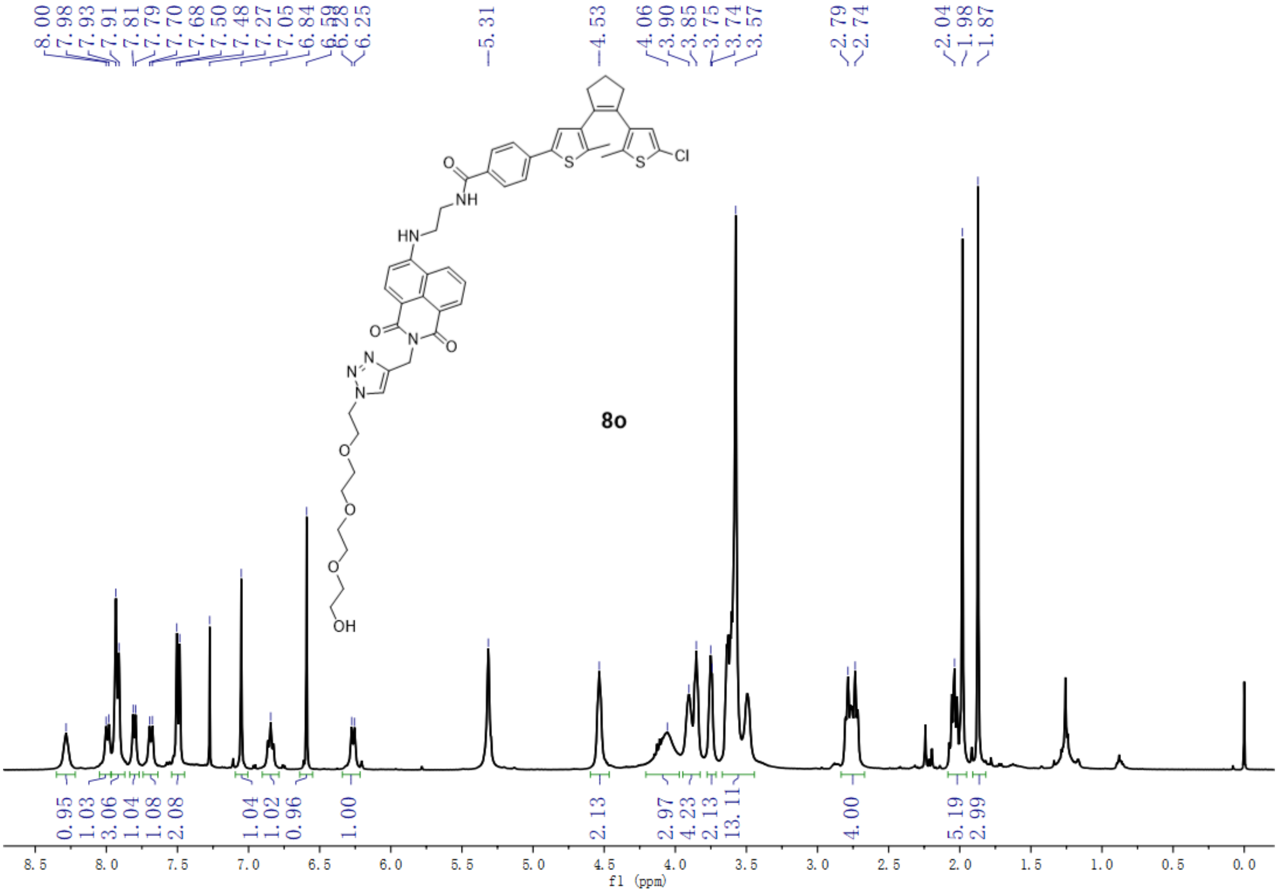

^{1}H NMR spectrum of compound **8o**

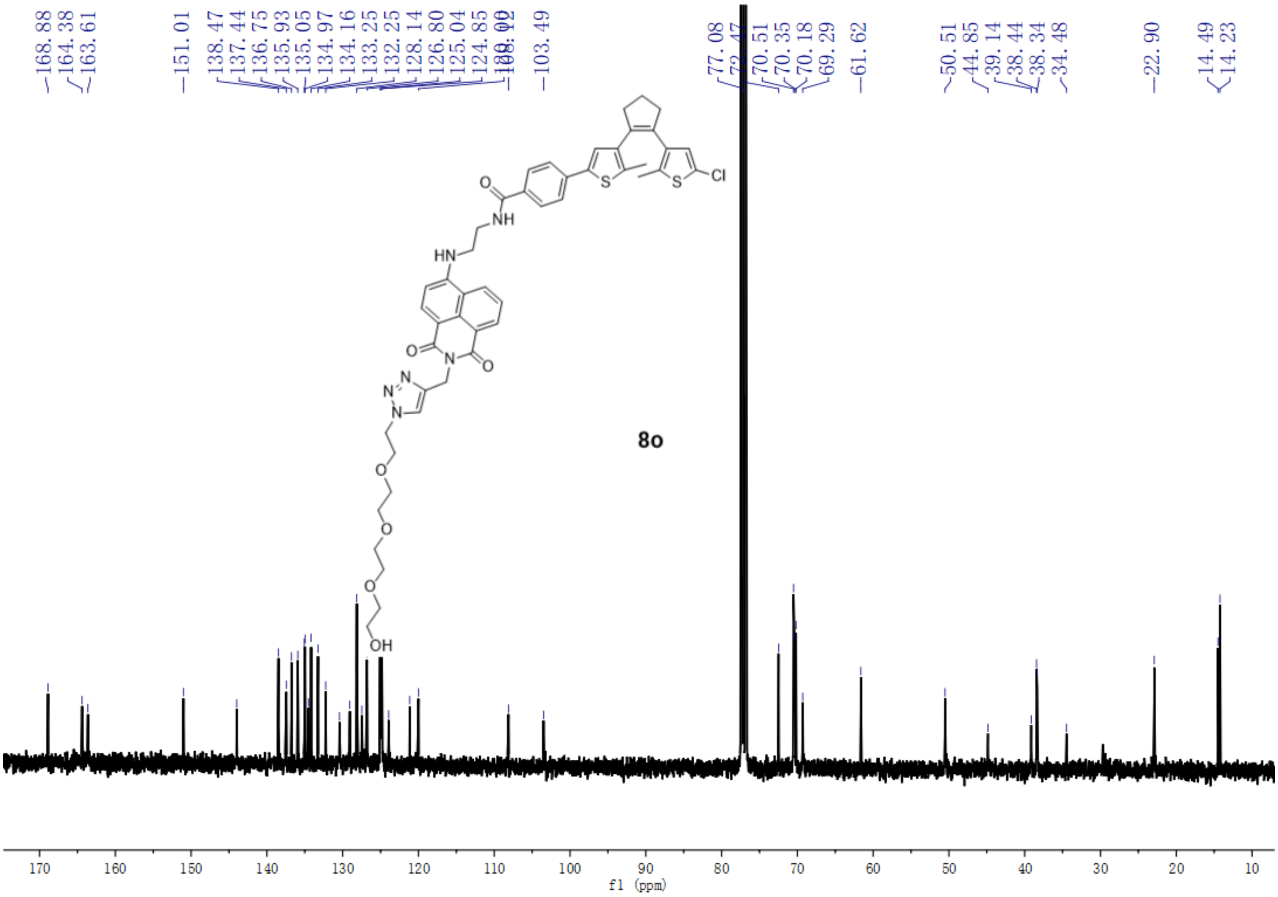

^{13}C NMR spectrum of compound **8o**

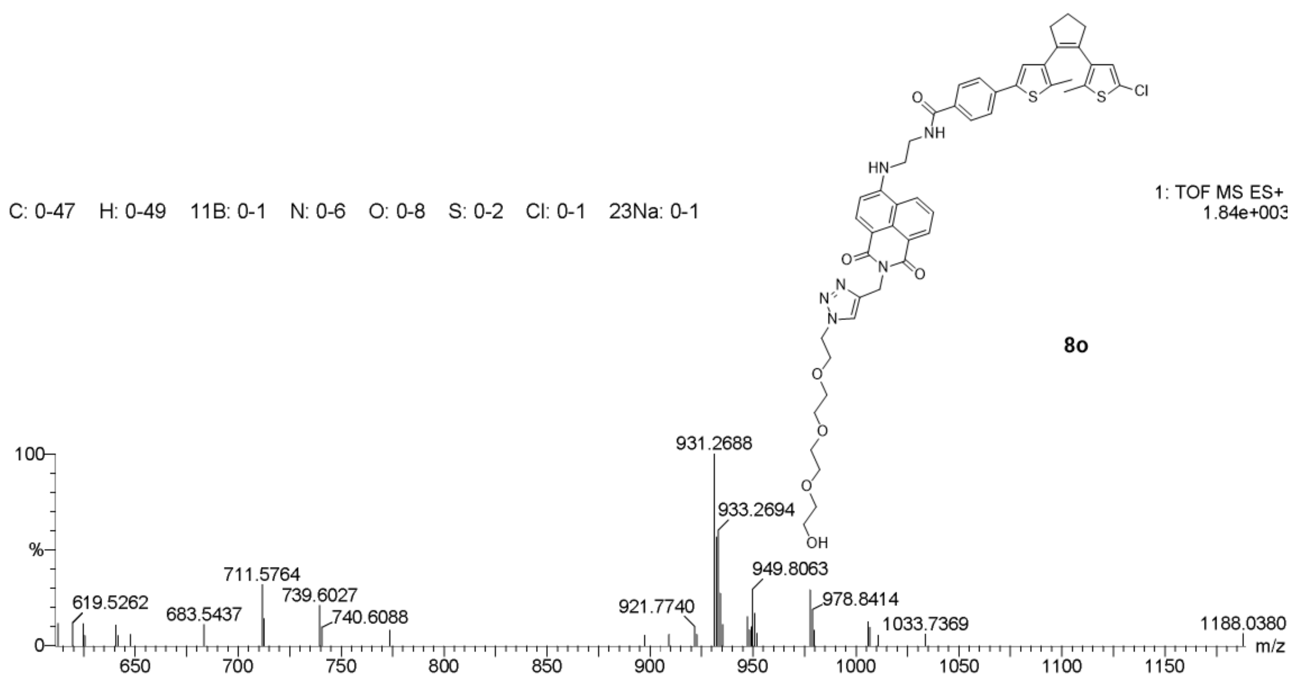

Minimum:				-1.5			
Maximum:		30.0	50.0	100.0			
Mass	Calc. Mass	mDa	PPM	DBE	i-FIT	i-FIT (Norm)	Formula
931.2688	931.2690	-0.2	-0.2	25.5	7.9	0.0	C47 H49 N6 O7 S2 Cl 23Na

HRMS spectrum of compound **8o**

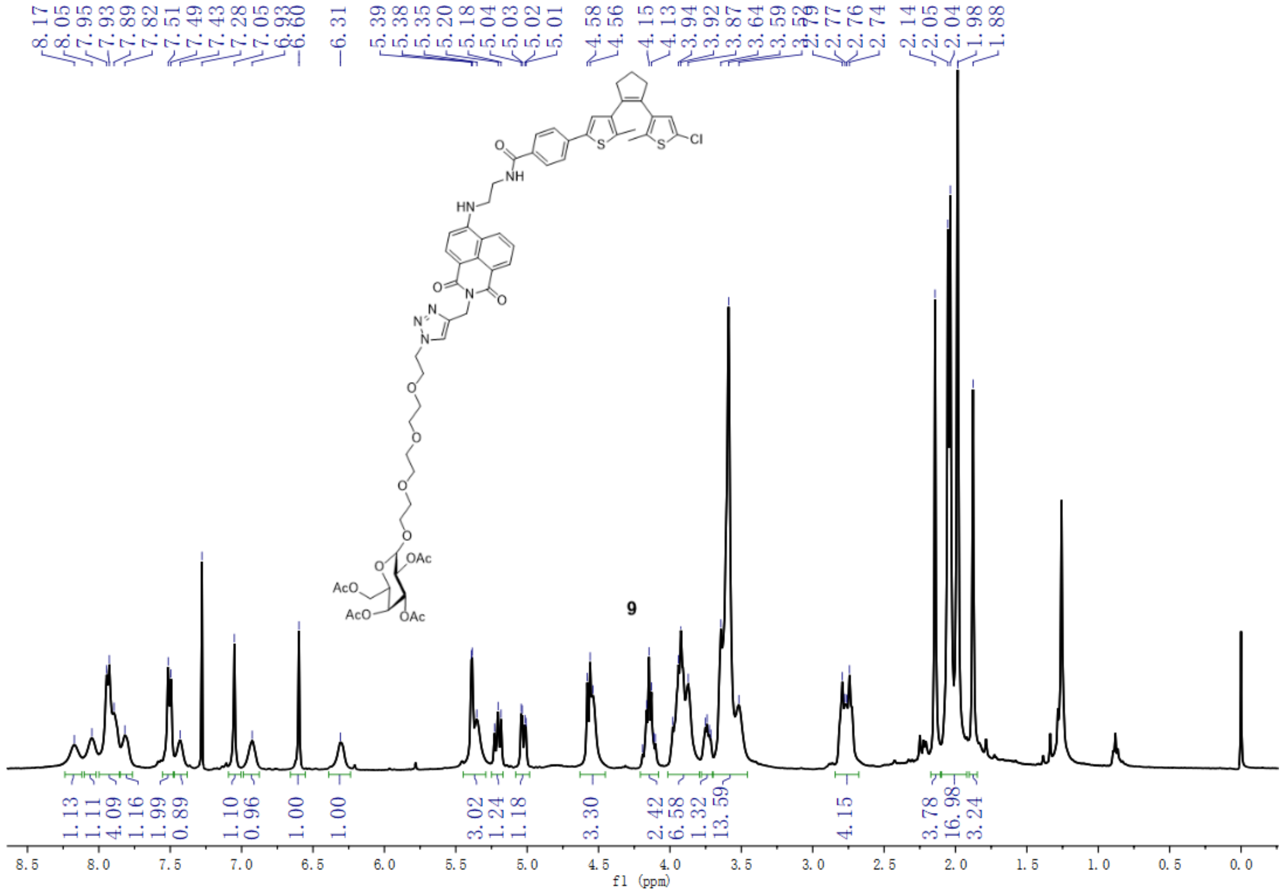

^{1}H NMR spectrum of compound **9**

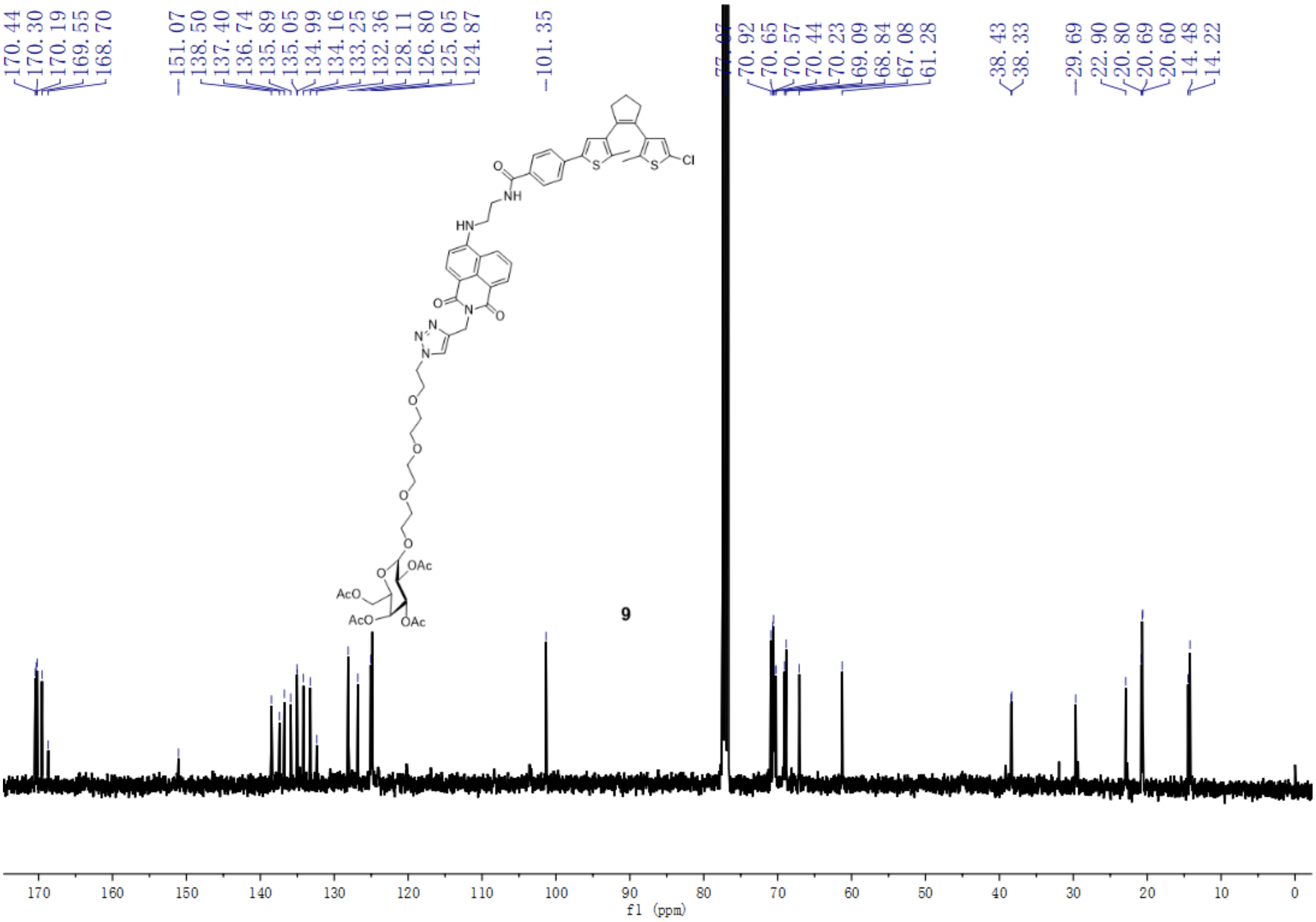

^{13}C NMR spectrum of compound **9**

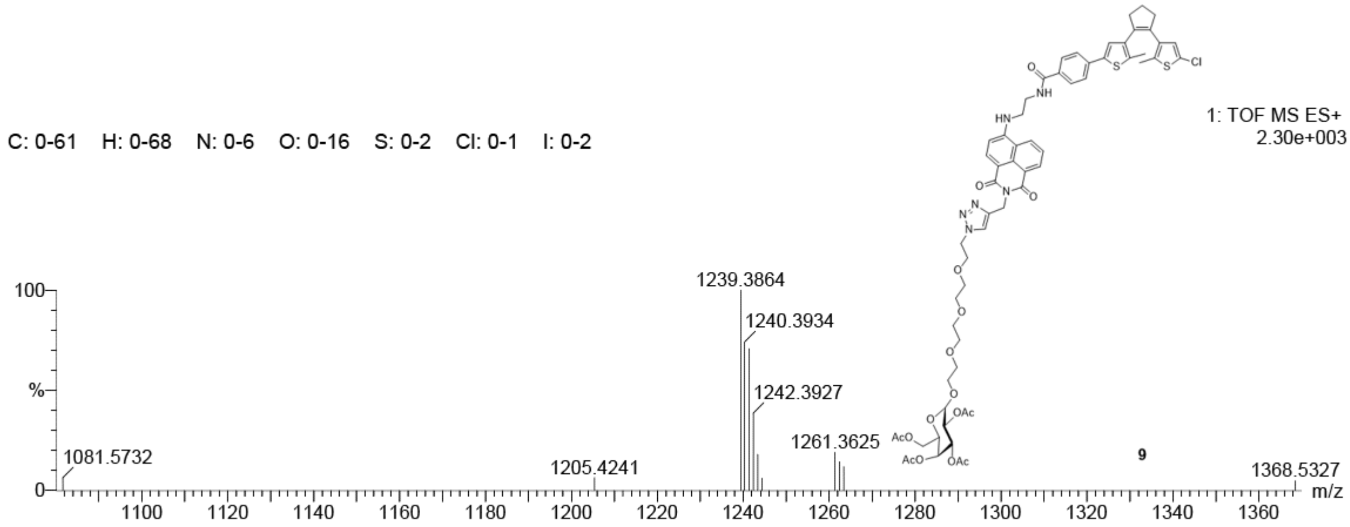

Minimum:				-1.5			
Maximum:		30.0	50.0	100.0			
Mass	Calc. Mass	mDa	PPM	DBE	i-FIT	i-FIT (Norm)	Formula
1239.3864	1239.3822	4.2	3.4	30.5	10.8	0.0	C61 H68 N6 O16 S2 Cl

HRMS spectrum of compound **9**

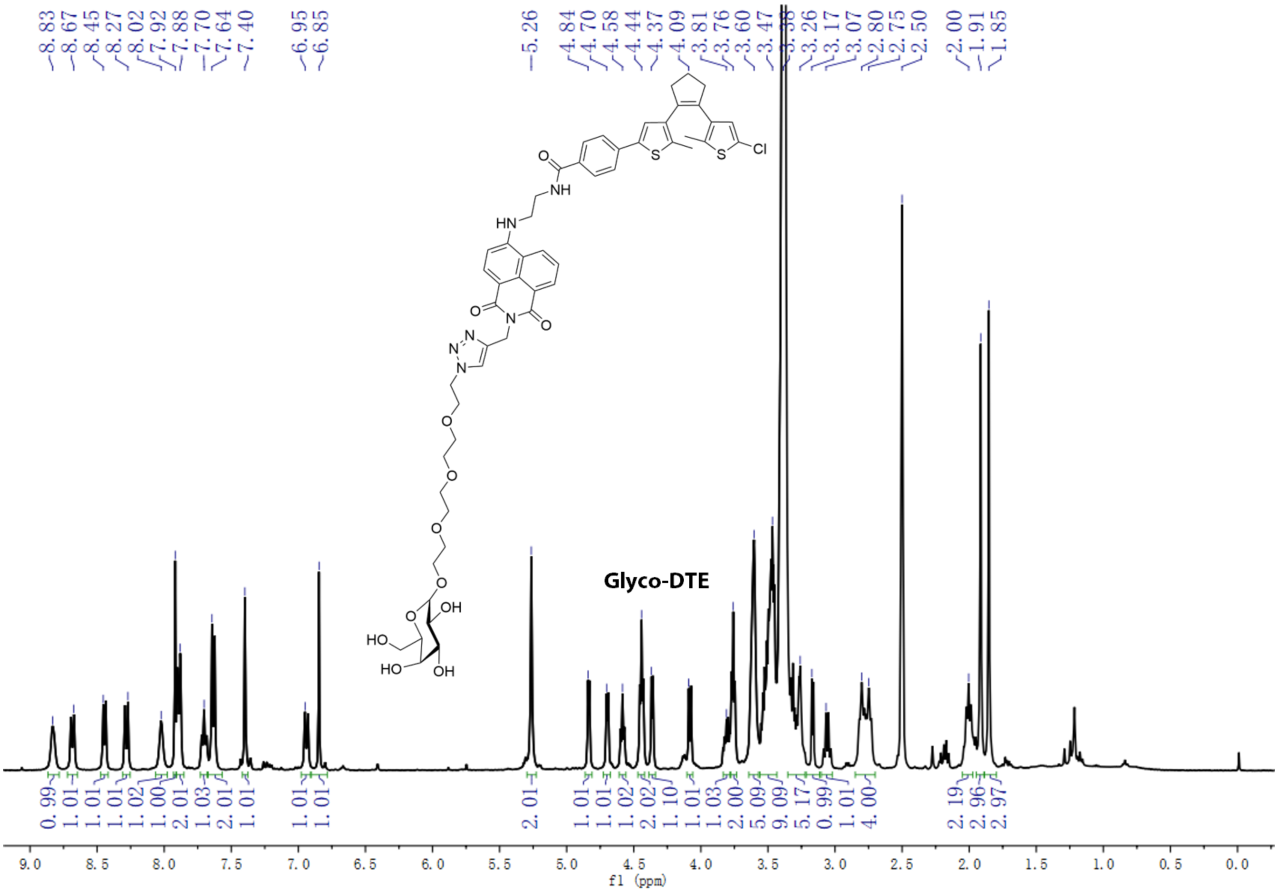

^{1}H NMR spectrum of compound **Glyco-DTE**

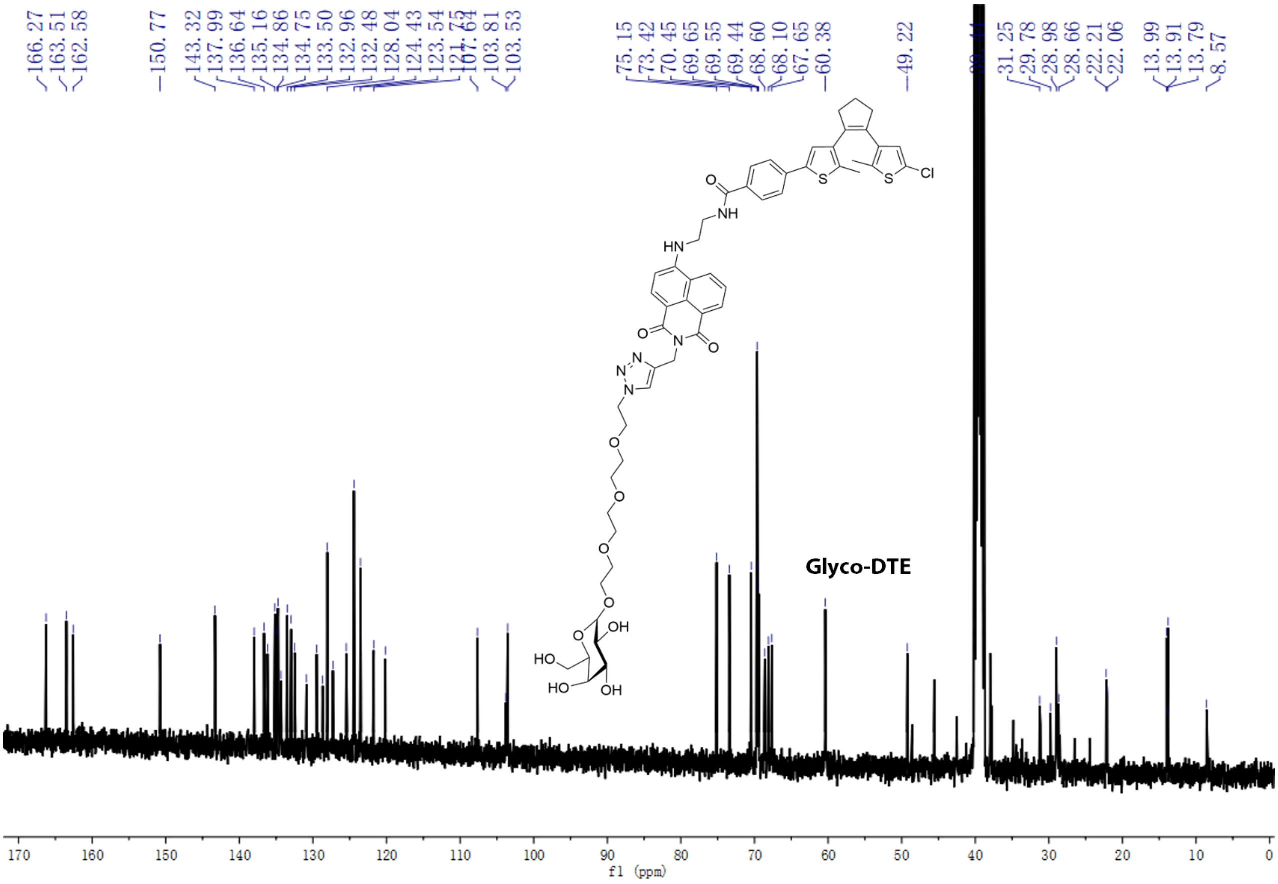

^{13}C NMR spectrum of compound **Glyco-DTE**

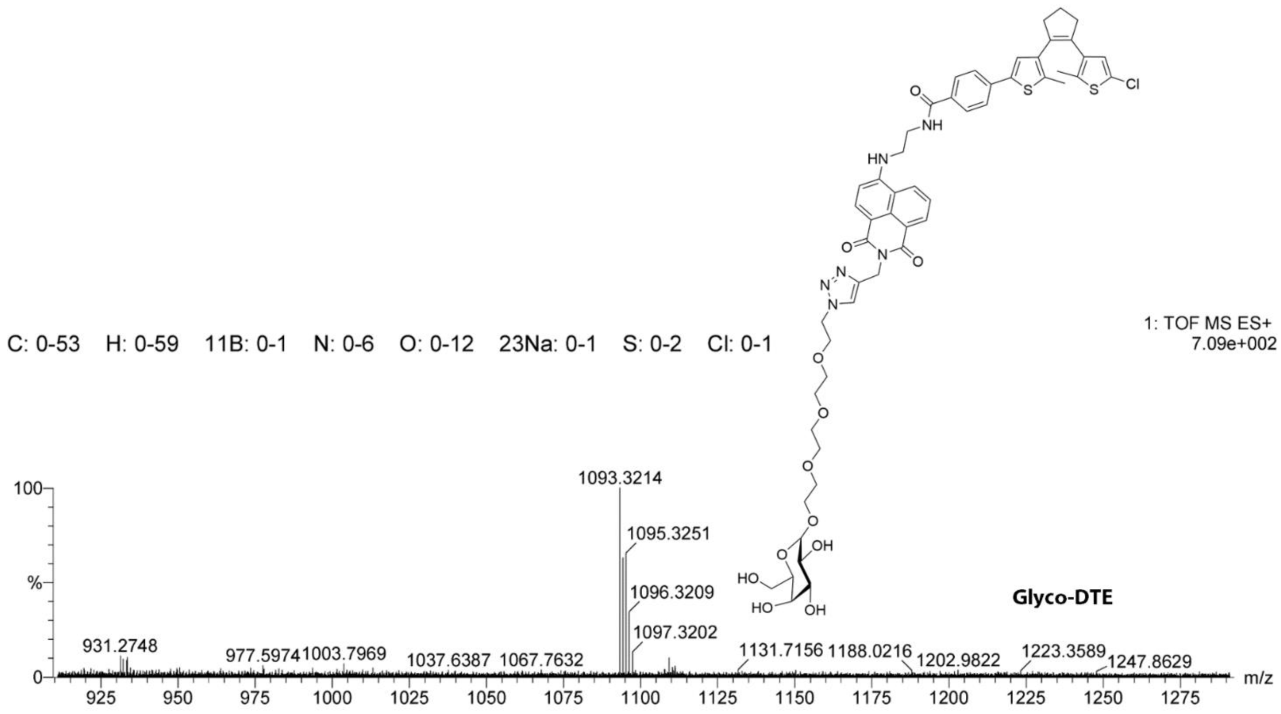

Minimum:				-1.5			
Maximum:		30.0	50.0	100.0			
Mass	Calc. Mass	mDa	PPM	DBE	i-FIT	i-FIT (Norm)	Formula
1093.3214	1093.3219	-0.5	-0.5	26.5	151.6	0.0	C53 H59 N6 O12 23Na S2 Cl

HRMS spectrum of compound **Glyco-DTE**
